# Enhanced brain tumor classification using graph convolutional neural network architecture

**DOI:** 10.1038/s41598-023-41407-8

**Published:** 2023-09-11

**Authors:** M. Ravinder, Garima Saluja, Sarah Allabun, Mohammed S. Alqahtani, Mohamed Abbas, Manal Othman, Ben Othman Soufiene

**Affiliations:** 1 CSE, Indira Gandhi Delhi Technical University for Women, New Delhi, India; 2 Department of Medical Education, College of Medicine, Princess Nourah bint Abdulrahman University, P.O. Box 84428, 11671 Riyadh, Saudi Arabia; 3 Radiological Sciences Department, College of Applied Medical Sciences, King Khalid University, 61421 Abha, Saudi Arabia; 4 BioImaging Unit, Space Research Centre, Michael Atiyah Building, University of Leicester, Leicester, LE1 7RH UK; 5 Electrical Engineering Department, College of Engineering, King Khalid University, 61421 Abha, Saudi Arabia; 6 PRINCE Laboratory Research, ISITcom, University of Sousse, Hammam Sousse, Tunisia

**Keywords:** Diseases, Health care

## Abstract

The Brain Tumor presents a highly critical situation concerning the brain, characterized by the uncontrolled growth of an abnormal cell cluster. Early brain tumor detection is essential for accurate diagnosis and effective treatment planning. In this paper, a novel Convolutional Neural Network (CNN) based Graph Neural Network (GNN) model is proposed using the publicly available Brain Tumor dataset from Kaggle to predict whether a person has brain tumor or not and if yes then which type (Meningioma, Pituitary or Glioma). The objective of this research and the proposed models is to provide a solution to the non-consideration of non-Euclidean distances in image data and the inability of conventional models to learn on pixel similarity based upon the pixel proximity. To solve this problem, we have proposed a Graph based Convolutional Neural Network (GCNN) model and it is found that the proposed model solves the problem of considering non-Euclidean distances in images. We aimed at improving brain tumor detection and classification using a novel technique which combines GNN and a 26 layered CNN that takes in a Graph input pre-convolved using Graph Convolution operation. The objective of Graph Convolution is to modify the node features (data linked to each node) by combining information from nearby nodes. A standard pre-computed Adjacency matrix is used, and the input graphs were updated as the averaged sum of local neighbor nodes, which carry the regional information about the tumor. These modified graphs are given as the input matrices to a standard 26 layered CNN with Batch Normalization and Dropout layers intact. Five different networks namely Net-0, Net-1, Net-2, Net-3 and Net-4 are proposed, and it is found that Net-2 outperformed the other networks namely Net-0, Net-1, Net-3 and Net-4. The highest accuracy achieved was 95.01% by Net-2. With its current effectiveness, the model we propose represents a critical alternative for the statistical detection of brain tumors in patients who are suspected of having one.

## Introduction

A group of aberrant brain cells, whether malignant or not, is referred to as a Brain Tumor (BT). Brain tumors may or may not be cancerous but nevertheless, they cause serious problems in the skull region because of the enormous pressure it puts on the cranium due to lack of space in the skull. Primary symptoms of BT being headache and dizziness. Brain Tumors are basically of two types: Primary and secondary types^1^. Primary tumors are those that start in the brain region itself, whereas secondary tumors begin in another part of the body and spread to the brain region to develop into a brain tumor. Although brain tumors are generally lethal, but the severity and the prognosis of the disease depends upon factors like Family history, Age and Race etc. Nevertheless, BT continues to be one of the top diseases that claim thousands’ lives every year. Therefore, early and accurate detection of brain tumor is extremely crucial for proper treatment and better survival rate. Many techniques ranging from simple ML classifications of K-Nearest Neighbors (KNN), K-means and Support Vector Machine (SVM) to state-of-the-art techniques like Deep Neural Networks (DNN) and CNN have been employed in the past with decent accuracy score and recall. However, accurate tumor classification with low bias and low variance on a generalized model continues to be a field of active research. BT is a pretty lethal disease with a grim survival rate. According to American Cancer Society carried out a study and found out that about 23,820 malignant tumors were detected out of all the brain tumors detected in the US in 2019.It was further stated that about 17,760 of these patients die from the brain cancer. However, the survival rates vary depending upon the type of tumor^2^. Since it is well known that brain tumors are an unnatural development of cells in the cerebral region, there have been innumerous studies and ways to classify these brain tumors. Brain tumor classification is an important topic of research, and it continues to attract a lot of researchers worldwide to present better and better classification models still. This paper's primary contributions are:

1. Explore the magnetic resonance imaging (MRI) dataset as graphs and application of neural networks on it.
2. Combining the CNN and GNN to make a state-of-the-art graph convolutional network (GCN) to yield accurate and precise classification results.
3. Five novel neural networks namely Net0, Net1, Net2, Net3, and Net4 are designed for brain tumor classification and investigate which network yields best accuracy.

In this research, we try to realize this relation awareness for the identification and categorization of brain tumors by considering the MRI images as a graph data structure and following a series of modelling steps which are discussed in greater details in the subsequent sections of the paper. This study's goal is to create a unique method for improving the precision of brain tumor diagnosis utilizing a combination of conventional CNN to learn image-level features and GCN to learn relation-aware representation features. The outcome is that the combination CNN and GCN networks will provide better performance compared to working alone. The paper presents a novel CNN and GCN combination network to achieve more accurate diagnoses.

The remaining sections of the paper are structured as follows: Section "Related work" presents an overview of Related Works, while Section "Materials and methods" delves into the Materials and Methods used in our research. In Section "Proposed approach and implementation", we introduce our proposed approach and describe its implementation. Section "Result and discussion" is dedicated to discussing the obtained results, and finally, Section “Conclusions” concludes the paper.

## Related work

In order to automatically classify brain malignancies using brain magnetic resonance images, Mohsen et al.^2^ employed the deep learning approach and evaluated the results. To train the Deep Neural Network classifier for the categorization of brain tumor, a collection of features was retrieved from the segmented brain MRI images using the Discrete Wavelet Transform (DWT) feature extraction technique. Using brain MRI pictures, the tumor was divided into four groups: metastatic bronchogenic carcinoma tumor, glioblastoma, sarcoma, and normal brain MRI tumor. Data was first acquired, then image segmented using fuzzy C-means, feature extracted using DWT and Principal component analysis (PCA), and finally tumor classification using DNN.

For the automatic diagnosis, identification, and location of brain tumor, Abd-Ellah et al.^3^ presented a two-phase paradigm. The system structure's first component includes preprocessing, CNN-based feature extraction, and feature classification (Error Correcting Output Codes—SVM) method. The first stage of the system identifies brain tumor by categorizing MRIs as either normal or abnormal, while the second stage focuses on localizing the tumor spot utilizing a fully developed five-layer Region based Convolutional Neural Network (RCNN).

Lakshmi and Hemalatha^4^, proposed a two stage Computer Aided System (CAD) that automatically finds out and classifies the BT into different classes using MRI images. In the first stage, the image is classified as either normal or abnormal images and in the next stage, they are classified into Malignant (Cancerous) and Benign (Non-Cancerous) tumors. The CAD works on feature extracted using feature Extraction techniques like PCA and DWT then SVM is applied on it.

Goswami and Bhaiya^5^, proposed a hybrid algorithm called “Hybrid Abdominal Detection Algorithm”. This is used to find the irregularities in any area of the body using MRI images.

Casamitjana et al.^6^, introduced a framework of machine learning for detection and classification of Brain Tumors in MRI data and the features were extracted using PCA and DWT.

Saltz et al.^7^ utilized a hierarchical method to segment brain tumors in MRI images. They applied a hierarchical CNN on the dataset and evaluated the performance using the metrics named Dice Score Coefficient, Positive Predictive Value, and Sensitivity.

In^8^ Arakeri and Reddy describes an ensemble classifier-based computer-aided diagnosis (CAD) system to identify benign or malignant brain tumors using MRI. The proposed segmentation technique was used to automatically extract brain tumor tissue from MR images. The texture, form, and border properties of a tumor are extracted and used to represent it. The most significant features are identified through the application of ranking features based on information acquisition and the use of independent component analysis methods. To characterize the tumor, these attributes are then utilized to train an ensemble classifier that includes an SVM, an artificial neural network, and KNN classifier. Pereira et al.^9^ used a fully convolutional 3D method. The expansion of 2D-CNN to 3D poses substantial problems, including an increase in the number of parameters as well as large memory and computing demands. These and other essential design concerns, including the network's depth, the training sampling technique, and the fully convolutional method is utilized to get dense inference.

Swati et al.^10^ uses CNN with transfer learning to employ intelligent feature extraction and feature representations. Abiwinanda et al.^11^ proposed HKFCM-CNN, Hybrid KFCM-CNN that uses Fuzzy K means Clustering using Kernels for automatic tumor classification. The Hybrid KFCM approach is used to separate a tumor region from an MRI brain picture. Seetha et al.^12^ proposed a method by using T1-weighted contrast enhanced MRI images, researchers built a CNN model for brain tumor classification. They accomplished this in two major steps. The photos were first preprocessed using several image processing techniques, and then CNN was used to classify them. This study used a dataset of 3064 photos that included three types of brain cancers: glioma, meningioma, and pituitary tumors. The greatest testing precision was discovered to be 93.33 percent, with an average precision of 93.33 percent.

Othman and Basri^13^, propose an automated identification of brain tumors using Convolutional Neural Networks (CNN) classification. The model makes use of small kernels to implement the deeper architecture while the neuron weights were kept small. Upon finding the results, it was found that the model received an accuracy of about 97.5% with a much lower complexity when compared to other state of the art methods.

Sachdeva et al.^14^ introduced a Probabilistic Neural Network approach that utilized data and image processing techniques for implementing brain tumor classification. The paper implemented the Probabilistic Neural Network method to achieve accurate classification of brain tumors. The decision-making process involved two steps: first, performing Feature Extraction using PCA, and then providing the extracted features to the Probabilistic Neural Network for further classification.

Deepak and Ameer^15^, proposed a collection of contrast T1-weighted MR images from was used to create a multiclass brain tumor classification system. Primary brain tumors like (AS), (GBM), (MED), (MEN), (MET), and normal regions, were photographed (NR). In this study, a content-based active contour model is employed to extract 856 areas of interest (SROIs) from the data, followed by the retrieval of 218 texture features. The dimensionality of the feature space is reduced using PCA. Artificial Neural Networks (ANN) is then utilized to classify the six groups, resulting in the PCA-ANN method. Three different sets of experiments are carried out, with the first one evaluating the precision of the ANN technique for classification. The second experiment involves using a PCA-ANN technique with random sampling.

Sajjad et al.^16^, focuses on the classification of brain tumors into three main classes, namely glioma, meningioma, and pituitary tumors. The authors have used GoogleNet to extract features from the brain MRI images. After successful feature extraction, a series of classifiers were applied on the extracted features along with fivefold cross validation. The model accuracy was found out to be 98%.

Mehrotra et al.^17^ proposed a transfer learning approach for AI-based BT classification. They showed how pre-trained models can enhance classification performance with little labeled data. Ullah et al.^18^ introduced a hybrid image enhancement-based brain MRI images classification technique. They combined image enhancement methods with machine learning to achieve accurate tumor classification. Çinar and Yildirim^19^ developed hybrid CNN architecture for tumor detection on brain MRI images. The combination of convolutional layers and other techniques resulted in improved accuracy.

Diaz-Pernas et al.^20^ presented a deep learning approach utilizing a multiscale CNN for BT classification and segmentation. Their method showcased promising results in both classification and localization tasks. Raja^21^ proposed a BT classification method using a hybrid deep autoencoder with Bayesian fuzzy clustering-based segmentation. The integration of autoencoders and clustering techniques enhanced classification performance. Polat and Güngen^22^ explored the use of deep transfer learning for BT classification from MR images. Their study demonstrated the potential of transfer learning in medical image analysis. Kaplan et al.^23^ investigated the application of modified local binary patterns (LBP) feature extraction methods for brain tumor classification. The modified LBP features contributed to improved classification accuracy. Kang et al.^24^ proposed an ensemble approach combining deep features and machine learning classifiers for MRI-based brain tumor classification. The ensemble technique yielded superior performance. Alzubaidi et al.^25^ introduced MedNet, a pre-trained CNN model specialized for medical imaging tasks. The MedNet model showcased potential for BT classification. Raza et al.^26^ presented a hybrid deep learning-based approach for BT classification, utilizing an ensemble of models to achieve improved results.

Lakshmi and Nagaraja Rao^27^ proposed a deep learning approach for BT MRI classification, showing promising results in the classification task. Ge et al.^28^ employed pair wise GANs to enlarge the training dataset for molecular-based brain tumor classification. The approach improved the model's generalization capability. Arif et al.^29^ developed a BT detection and classification system using a combination of biologically inspired orthogonal wavelet transform and deep learning techniques. Budati and Katta^30^ introduced an automated BT detection and classification system using machine learning techniques with IoT integration. Dehkordi et al.^31^ proposed a new evolutionary CNN for BT detection and classification, showcasing improved performance over traditional CNNs. Biratu et al.^32^ presented an enhanced region-growing method for BT MR image segmentation, which could contribute to improved classification performance. Ghassemi et al.^33^ combined deep neural networks with generative adversarial networks pre-training for BT classification based on MR images, demonstrating improved classification accuracy.

Recently, researchers have focused their attention^34^ to automating the feature extraction process and also standardizing the networks by exploring the scope of Convolutional Neural Networks and Transfer Learning in the field. All these techniques extract features of individual images in an automated fashion, but they lack the ability to learn the image level relationships or the pixel-to-pixel relationships. This creates a scope for adding a novel step in feature engineering that is to explore pixel-pixel relationships. The motivation and objective of this research is to devise a mechanism to account for the pixel-based relationships and to create a relation-aware representation for Brain tumor classification. Relation aware representation uses the relationships amongst the data points as a knowledge base for effective learning of the model.

## Materials and methods

### Dataset

**Figure 1:**
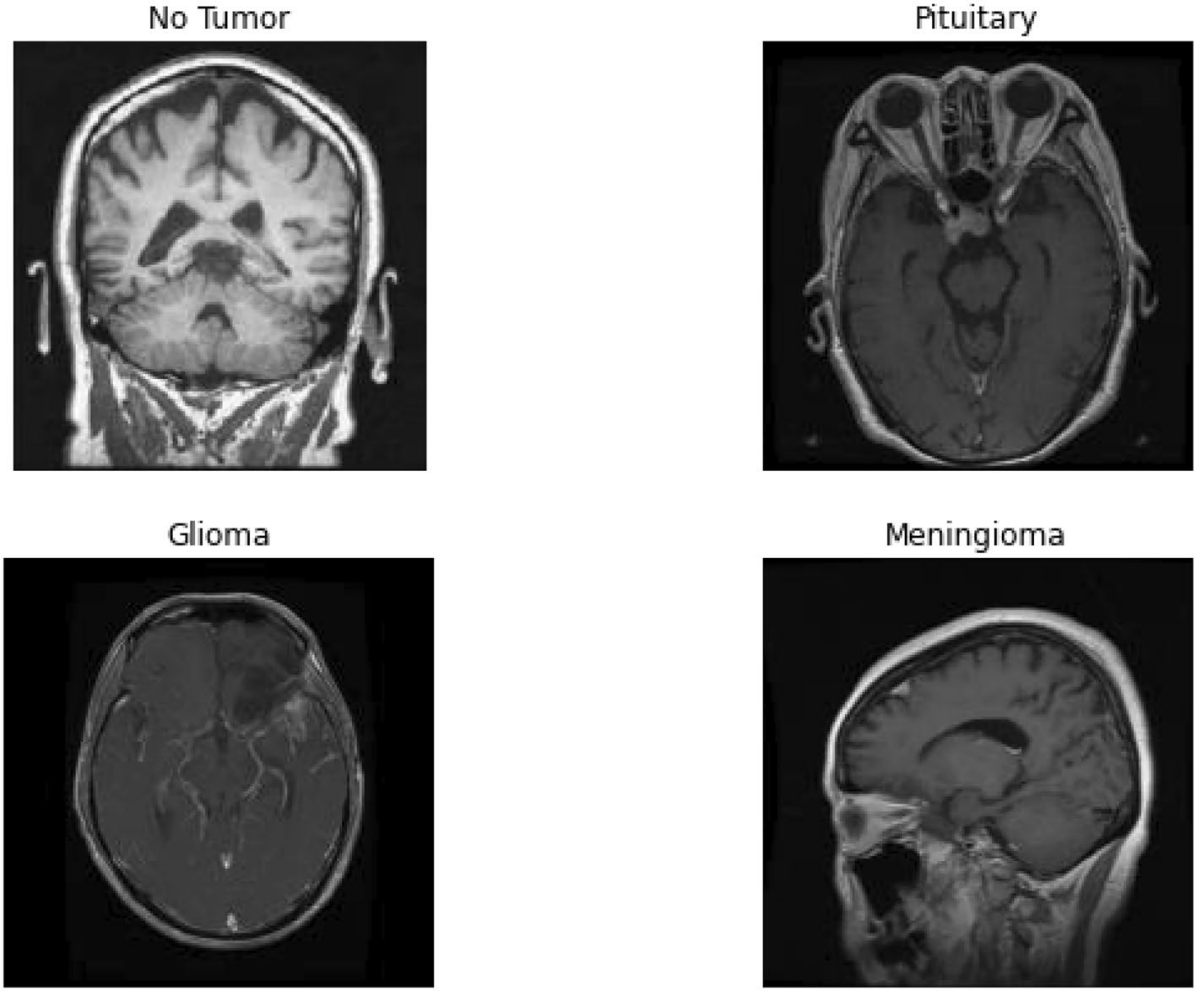
Brain tumor classes in the dataset.

The MRI image data has been taken from Kaggle which has about 3264 MRI images. The MRI provided in this data-set are a combination of T1, T2 and FLAIR types^35^ of different patients primarily classified into 4 categories, namely: No Tumor, Pituitary Tumor, Glioma Tumor, Meningioma Tumor and Sarcoma Tumor. Out of these 3094 images (512 × 512 × 3), 2700 images are put under the training folder and the rest 394 images are put under the testing folder. MRI stands for Magnetic Resonance. An MRI uses magnetic fields instead of x-rays, to generate elaborate images of the body part under observation. MRI is used for measuring tumor size in some cases. MRIs generate better and more detailed pictures than CT scans and therefore, are preferred over CT scans while diagnosing brain. For brain tumor detection, which can be either primary or secondary in its origin, the MRI can be done for brain and/or Spinal Cord depending upon the type of tumor. The dataset consists of 3094 brain MRIs which differentiate between tumorous and non-tumorous images. In Fig. 1, we have shown the images from all these classes in the dataset.

### Methods

The most popular and conventional methods used for detection and classification tasks are as follows and each of these techniques has some obvious setbacks which progressively take us to our approach.

#### Artificial neural network (ANN)

ANN is a structure motivated by the biological neural networks of the brain that is responsible for generating memory and thinking ability. In an artificial neural network, there are functional units called as “neurons” connected via weighted connections called as “weights”, we also have a constant threshold for adjusting the output that’s called as a “bias”. Like biological neural networks, we have multiple layers of these neurons connected to each other^36^.

**Figure 2:**
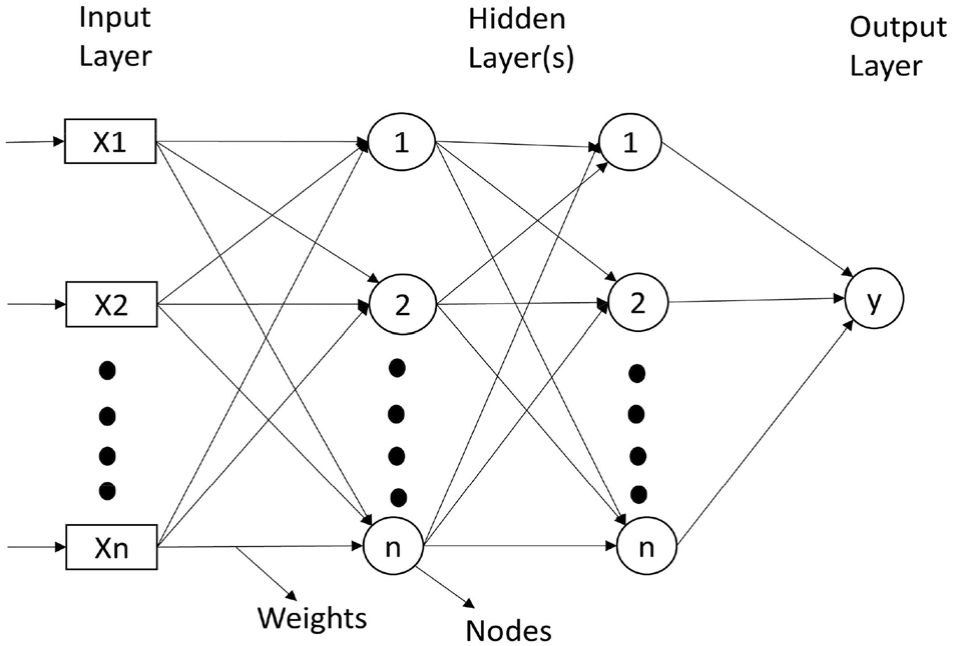
Layers in artificial neural network.

In a neural network (NN), layer 1 is the input layer, which includes all the input features. Layer 1 is followed by one or more hidden layers that aim to enhance the accuracy of the classification process. Input and output layers of the neural network are separated by the hidden layers. The number of hidden layers in a NN determines whether it is deep or not. A neural network with more than three hidden layers is referred to as a DNN. The structure of a NN is depicted in Fig. 2. For our problem of “Brain Tumor Classification”, if we use an artificial neural network, the following problems arise:

1. *Excessive Computation* If raw image dataset is fed into the neural network without proper feature extraction, the computation increases exponentially, as the feature vector would consist of all the pixels of the image where each pixel is considered to a feature of its own. This causes a lot of computation as the size of the feature vector increases dramatically as the number of pixels increases and all of these pixels have to be computed via the successive hidden layers which further increases the computation which is not practically possible with even the most sophisticated computing devices.
2. *Treats local pixels same as the pixels far apart* As there is no provision to find out the similarity between two pixels which belong to same region as same, ANN treats them as different pixels without considering the pixels in the same region under one group to reduce the computation efforts;
3. *Sensitive to location of an object in an image* The conventional ANN architecture does not have the provision to be independent of the location of tumor in the image and there is no way of storing the information about the tumor independent of the location;

Requires Feature Extraction: The conventional ANN architecture does not take care of feature extraction. The features need to be extracted manually. All of these issues can be solved using a CNN that is, convolutional neural networks.

#### Convolutional neural network (CNN)

CNN is a type of deep learning algorithm that takes in an image dataset and extract learnable features from it by assigning importance to the various aspects of the image. The Convolutional Neural Networks take care of the excessive computation for image dataset by series of Convolution and Pooling operations^37^. Various Convolutional filters are there that can detect different features in an image by just going over the pixel grid matrix with an appropriate stride. After convolving these pixel values with the filter, we get a feature map, this feature map might have some negative values in some places, to remove these negative values and to introduce non-linearity in the model, and we apply ReLu function on the feature map values. The Convolution steps might reduce the dimension of the original image matrix if valid padding is there (no padding), but this is not sufficient, and the computation required to carry out image classification process is still exponential. To reduce the dimensionality of the feature map further, we use another operation after Convolution that is called Pooling. Pooling operation carries out the process of dimensionality reduction in the feature map by the following process: First a 2 × 2 or 4 × 4 or any suitable dimension matrix is chosen, and the feature map is iterated in this order of the matrix such that only maximum value out of the 2 × 2 space in the feature map is taken out. This is called Max pooling^38^. Similarly, average and min pooling is also there. There are various advantages of this Pooling layer operation like, reduces dimension and computation, Reduces Overfitting as the number of parameters are less, and Model is tolerant towards variations. This is then sent to a fully connected DNN. While Convolutional Neural Networks have some obvious advantages over their contemporaries, Convolutional Neural Networks lack the sense of relational awareness and its representation. We introduce the concept of Graph data structure to incorporate relational awareness.

**Figure 3:**
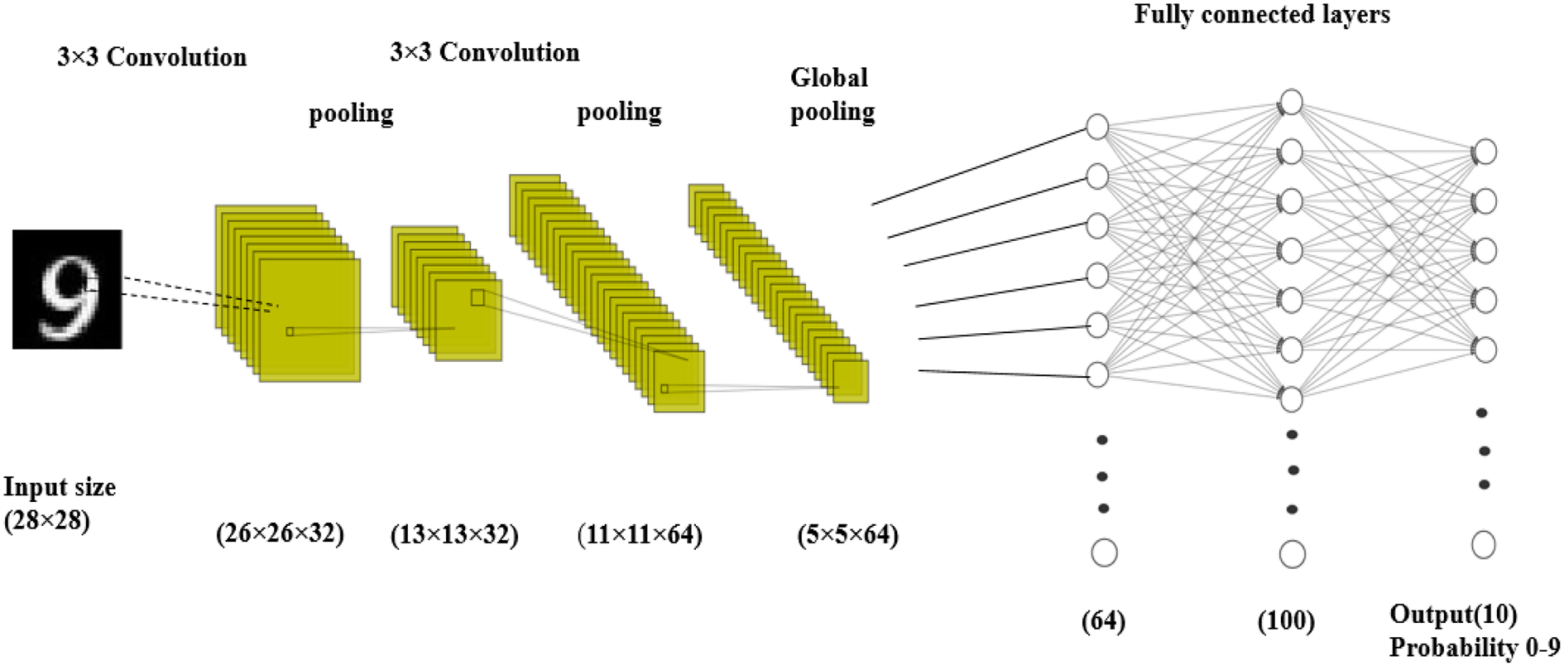
Layers in artificial neural network.

In Fig. 3, we have shown example architecture of a convolutional neural network:

#### Graph neural network (GNN)

**Figure 4:**
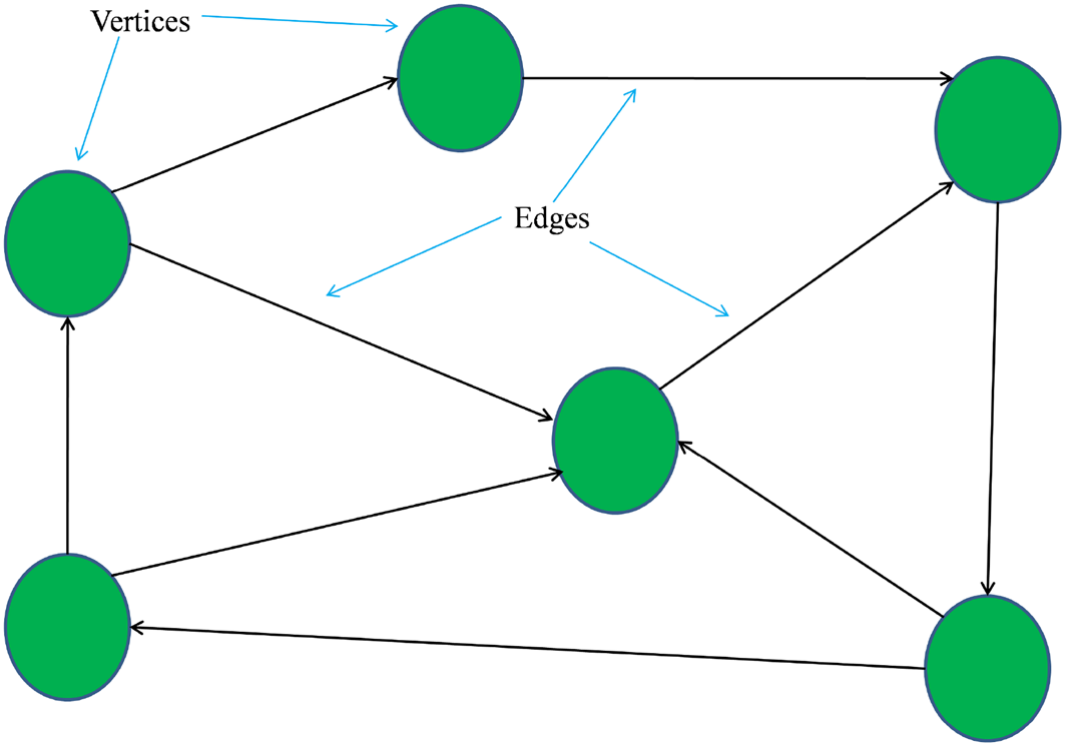
Directed graph.

A graph G can be defined as a set of nodes or vertices denoted by V and edges represented by E, such that G = (V, E). The edges may be directed or undirected. Figure 4 illustrates an example of a directed graph.

A graph is a very flexible data structure. It can be used to represent different real-life entities such as social media networks, molecules and even images. In our study, we use graph data structure to represent image dataset and then carry out different operations on it.

**Figure 5:**
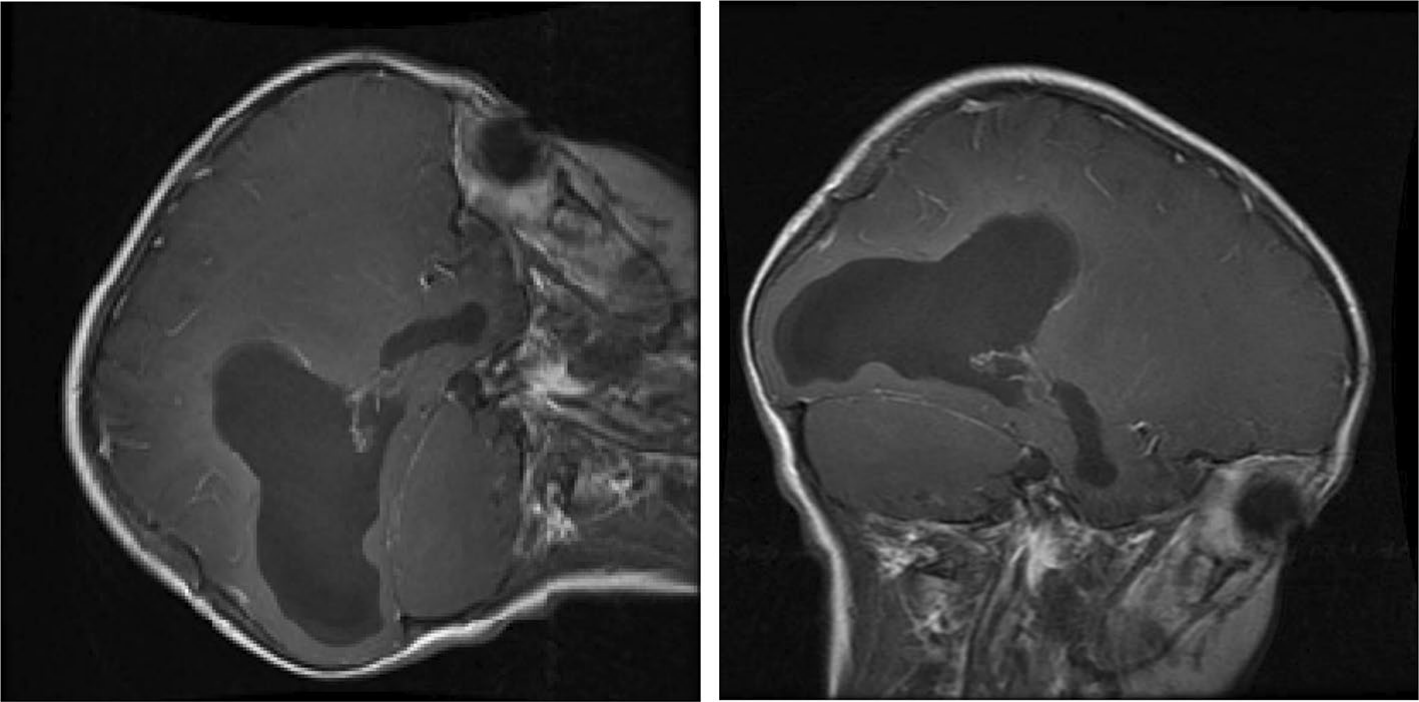
Isomorphism in graphs.

GNN belongs to a class of deep learning algorithms which can be directly applied to the graph data and carry out node level or edge level prediction tasks. Graph data is quite different than our conventional data that we feed into the neural networks generally, primarily because of the following reasons:

1. *Graph data is not definite in size* The dimension of a graph dataset might vary in the number of nodes. Thus, we require a neural network which can have arbitrary input dimension.
2. *Graphs are isomorphic* The graph data structure is isomorphic in nature meaning that the order of traversal of the graph can in fact change the entire image. Therefore, a single adjacency matrix is not sufficient for the graph representation. An example isomorphism in graph is as shown in Fig. 5.
3. *Graphs are non-Euclidean in nature* This means that the distances in the graph are non-Euclidean in nature and have no fixed distance between them. Because of all these reasons mentioned above, application of conventional deep learning and machine learning operations are difficult on the graph data structure. GNNs are part of representation learning which can effectively deal with all these issues faced by the graph data structure in deep learning. Graph Neural Network has nodes which can communicate with each other and share information with each other about them. The graph works on the concept of node embeddings such that the nodes are mapped to a d-dimensional embedding space which is low dimensional space instead of the real dimension of the graph under consideration. This way the similar nodes are embedded close together. This way the problem of pixel similarity is resolved using graph neural network. Consider a and b as two nodes of our graph^39^. X_a_ and X_b_ are two feature vectors corresponding to these nodes. These feature vectors are then passed through encoders, these encoders then convert the original features into the embeddings which are grouped together based upon the similarity in the features. There are different ways of finding these embeddings: Locality (local network neighborhoods), Aggregate, and Stacking multiple layers. Generally, we use Aggregate information as node embeddings.

## Proposed approach and implementation

We present a new method for identifying and categorizing brain tumors using an MRI dataset, which is based on a graph CNN model. The proposed method involves a series of networks for tumor classification and detection, and a comparative analysis is conducted among these networks. The conventional CNN model used to classify Brain Tumors does not define similarity between the local pixels. This is the problem because of the isomorphic nature of graphs (image). To solve this problem, we use a novel CNN based Graph Neural Network (GNN) that takes care of the similarity between local pixels by creating node embeddings for them. This model is a Graph based Convolutional Neural Network.

### Proposed model

In this study, we propose a novel approach that combines GNN and CNN to classify brain tumors into different classes. The conventional image representation as an n × n matrix of pixels presents certain drawbacks when training machine learning or deep learning models. Existing conventional models for Brain Tumor classification lack the capacity to retain and utilize pixel-related information for future classifications. Notably, nearby pixels are more likely to share similar properties and belong to the same class, whereas distant pixels may differ significantly. While this might resemble a problem of conventional image segmentation or clustering, both methods have significant drawbacks and limitations.

**Figure 6:**
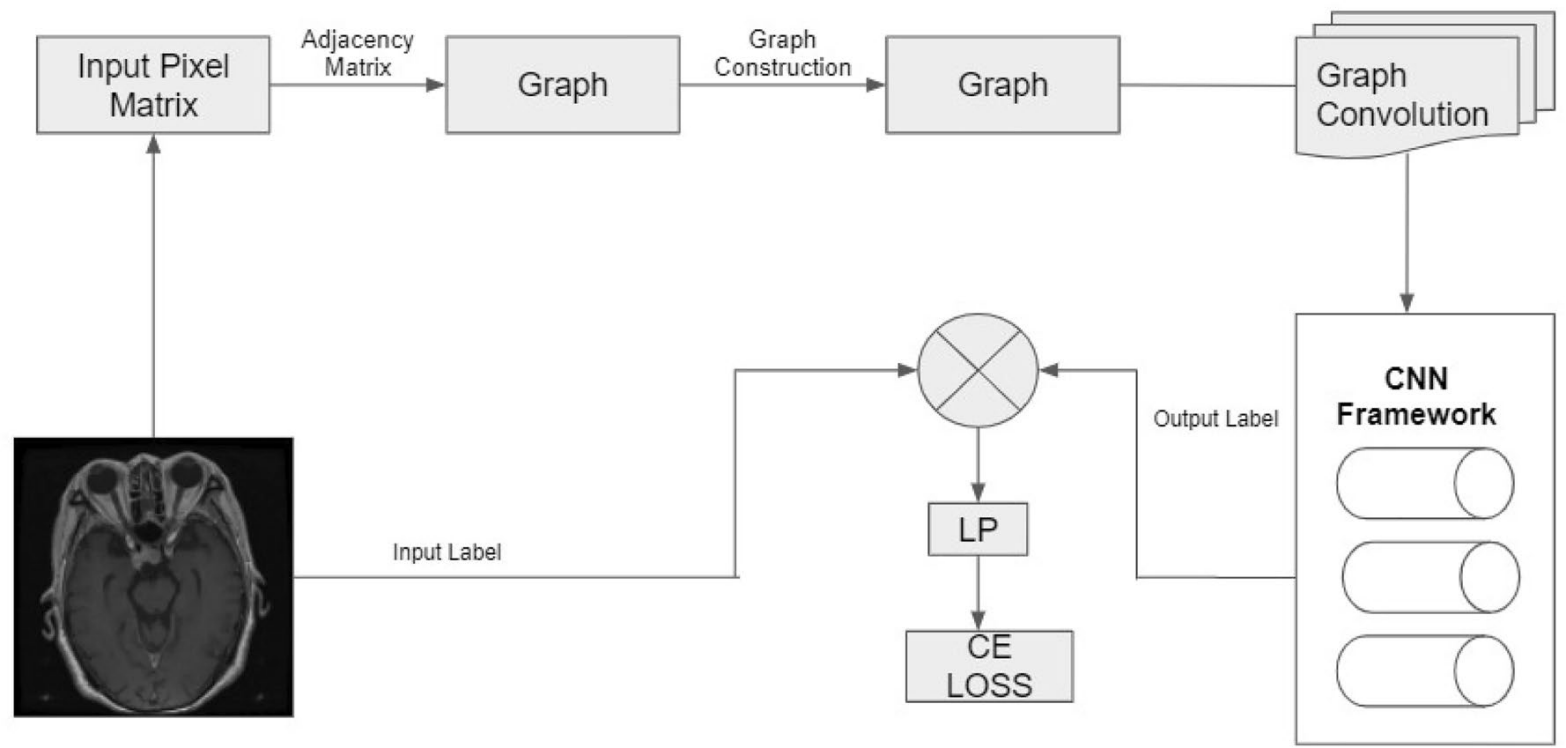
Proposed approach.

The proposed model's approach, illustrated in Fig. 6, comprises the following components:

1. Data pre-processing.
2. Generating a standard pre-computed weighted adjacency matrix kernel.
3. Overlaying this kernel on all training and testing images.
4. Incorporating relational awareness through the averaging operator, considering 'n' specified neighbors for each pixel (Graph Convolution operator).
5. Feeding this updated matrix through a vanilla Convolution Neural Network with 26 layers.

**Figure 7:**
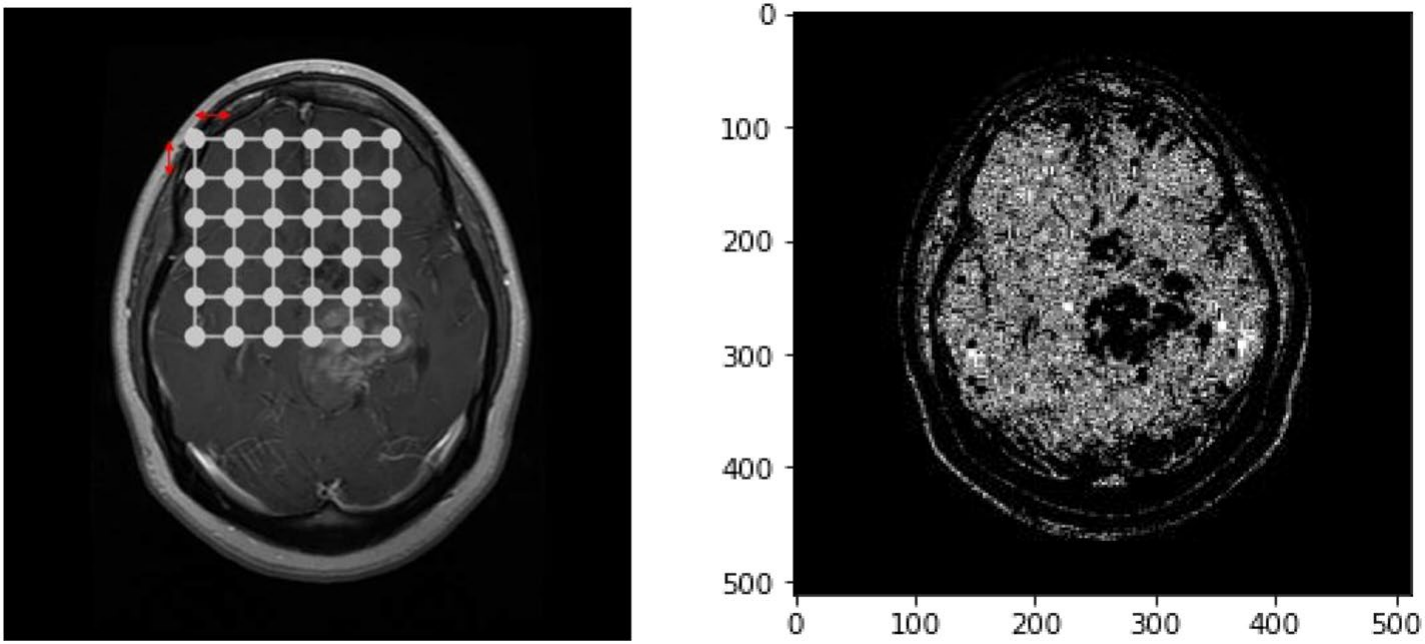
Example output of clustering (left) & Segmentation (right) algorithms without considering Euclidean Distances.

**Figure 8:**
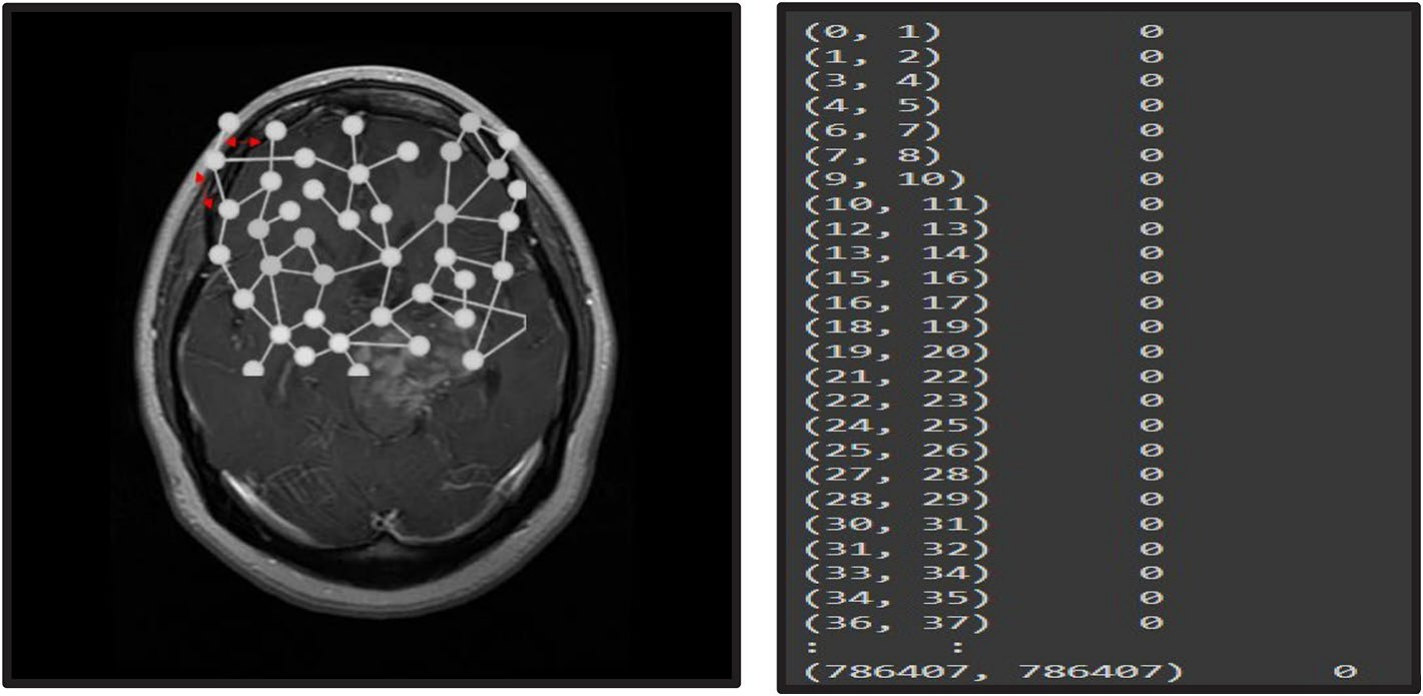
Example output of graph neural network (left) and graph depicting Euclidean distances (right).

The adjacency matrix in a Graph Convolutional Network (GCN) is constructed to represent the relationships between nodes in the graph. For an undirected graph with 'N' nodes, the binary adjacency matrix 'A' is an N × N matrix, where A_ij = 1 if there is an edge (connection) between nodes i and j, and A_ij = 0 if there is no edge. The vanilla CNN (Convolutional Neural Network) plays a crucial role in the Graph Convolutional Network (GCN) by serving as the downstream task network for the processed graph data. After applying the graph convolution operation to aggregate information from neighboring nodes and updating node features, the resultant graph data is fed into the vanilla CNN. The role of the vanilla CNN is to further process and extract hierarchical features from the graph-structured data, enabling the GCN to learn and represent complex patterns in the graph. Conventional clustering and segmentation algorithms typically rely solely on linear metrics such as Euclidean distances or Manhattan distances. However, in Fig. 7, we present an example of clustering and segmentation algorithms that do not take Euclidean distance into account. As a result, these conventional algorithms cannot perform random and non-Euclidean traversal of the image, leading to the omission of complete regional information between neighbors. Consequently, pixel similarity cannot be accurately measured using these methods. To address this limitation, we opt for a Graph data structure as a replacement for conventional image representation. Figure 8 illustrates the graph data structure as a network instead of a matrix, representing non-Euclidean distances.

### Implementation

This section presents the implementation of our proposed approach. The following five types of networks were designed for comparison purposes.

1. *Net-0 (Baseline Convolutional Neural Network (CNN))* This network has standard 15 layers of sequential Convolutional Neural Network without any added layers for Drop-out (DO) or Batch Normalization (BN);
2. *Net-1 (Graph based Convolutional Neural Network without Dropout (DO) or Batch Normalization (BN))* This network has standard 15 layers with a Graph based input with Graph Convolution Operator Operation;
3. *Net-2 (Graph based Convolutional Neural Network with Dropout (DO) or Batch Normalization (BN) with Gaussian Adjacency Matrix))* This network has standard 26 layers with Graph based input and Graph Convolution Operation along with Batch Normalization (BN) and Drop out (DO) with Gaussian Adjacency matrix**;**
4. *Net-3 (Graph based Convolutional Neural Network with Dropout (DO) or Batch Normalization (BN) with Uniform Adjacency Matrix)* This network has standard 26 layers with Graph based input and Graph Convolution Operation along with Batch Normalization (BN) and Drop out (DO) with Uniform Adjacency Matrix;
5. *Net-4 (Graph based Convolutional Neural Network with Dropout (DO) or Batch Normalization (BN) with Log normal Adjacency Matrix)* This network has standard 26 layers with Graph based input and Graph Convolution Operation along with Batch Normalization (BN) and Drop out (DO) with Log normal Adjacency Matrix.

**Figure 9:**
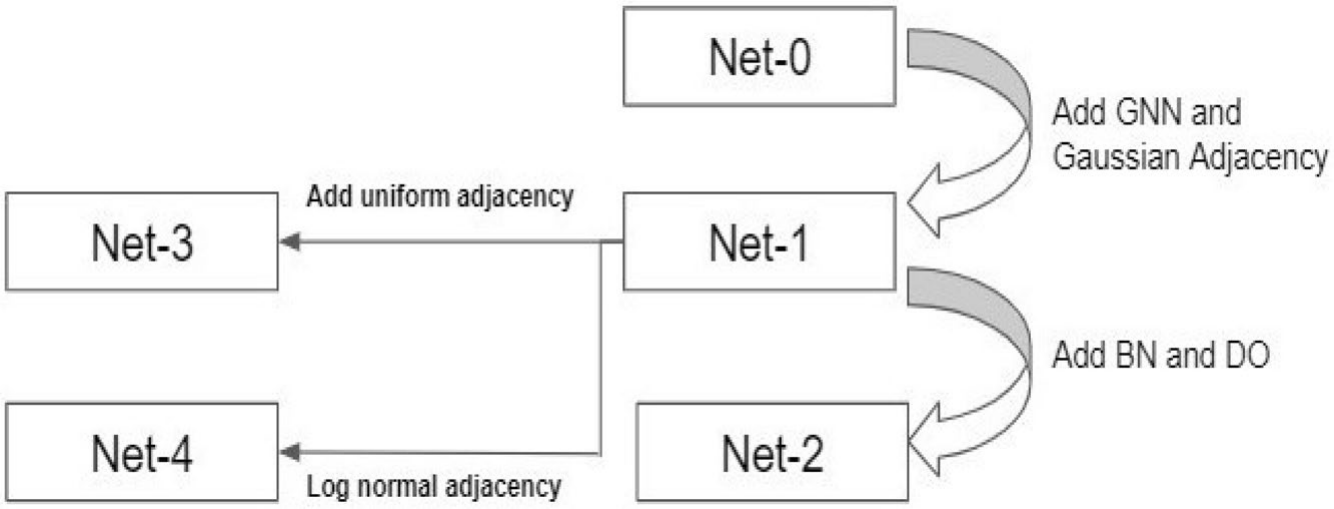
Relationship among the five types of networks.

Figure 9 below shows the relationship among the above five networks.

#### Data preprocessing and analysis

**Figure 10:**
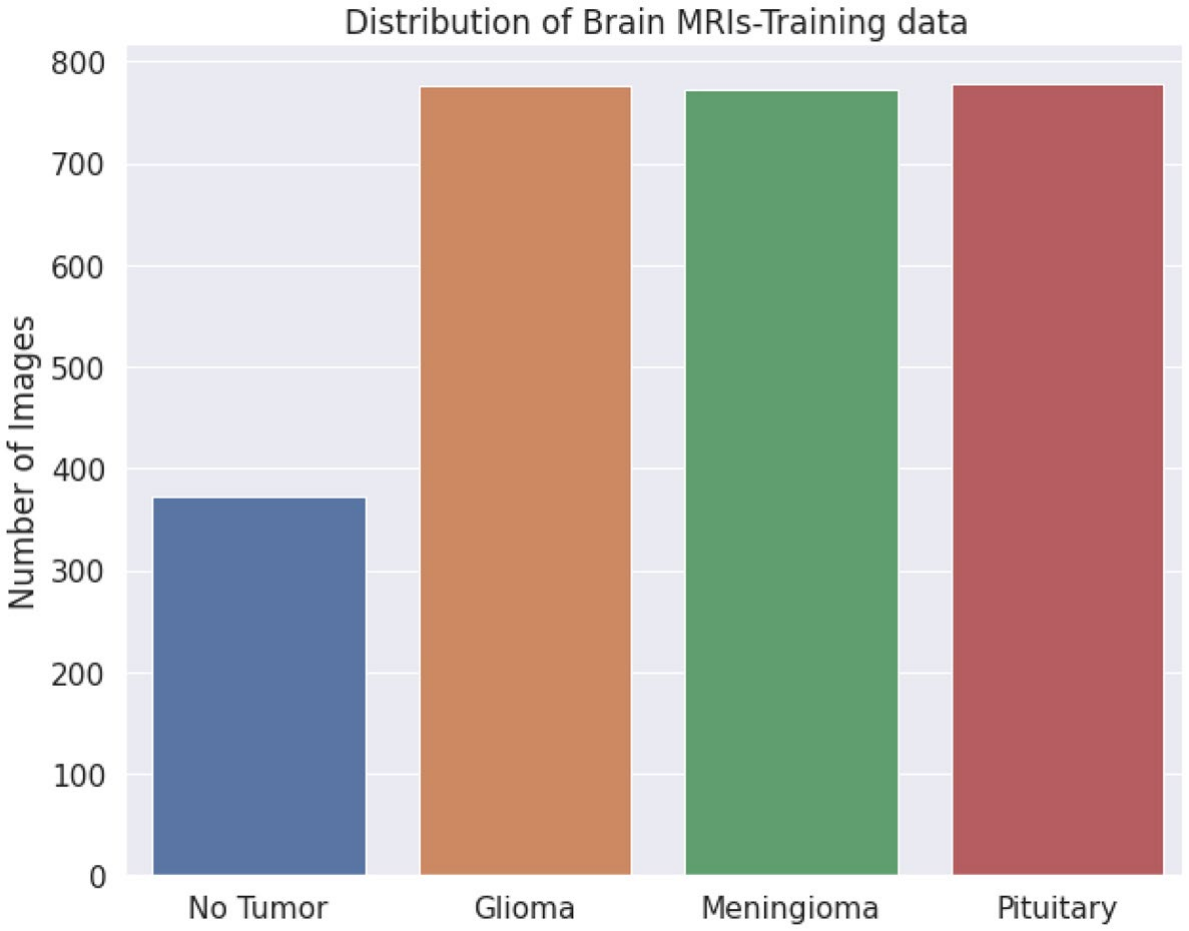
Distribution of training data.

**Figure 11:**
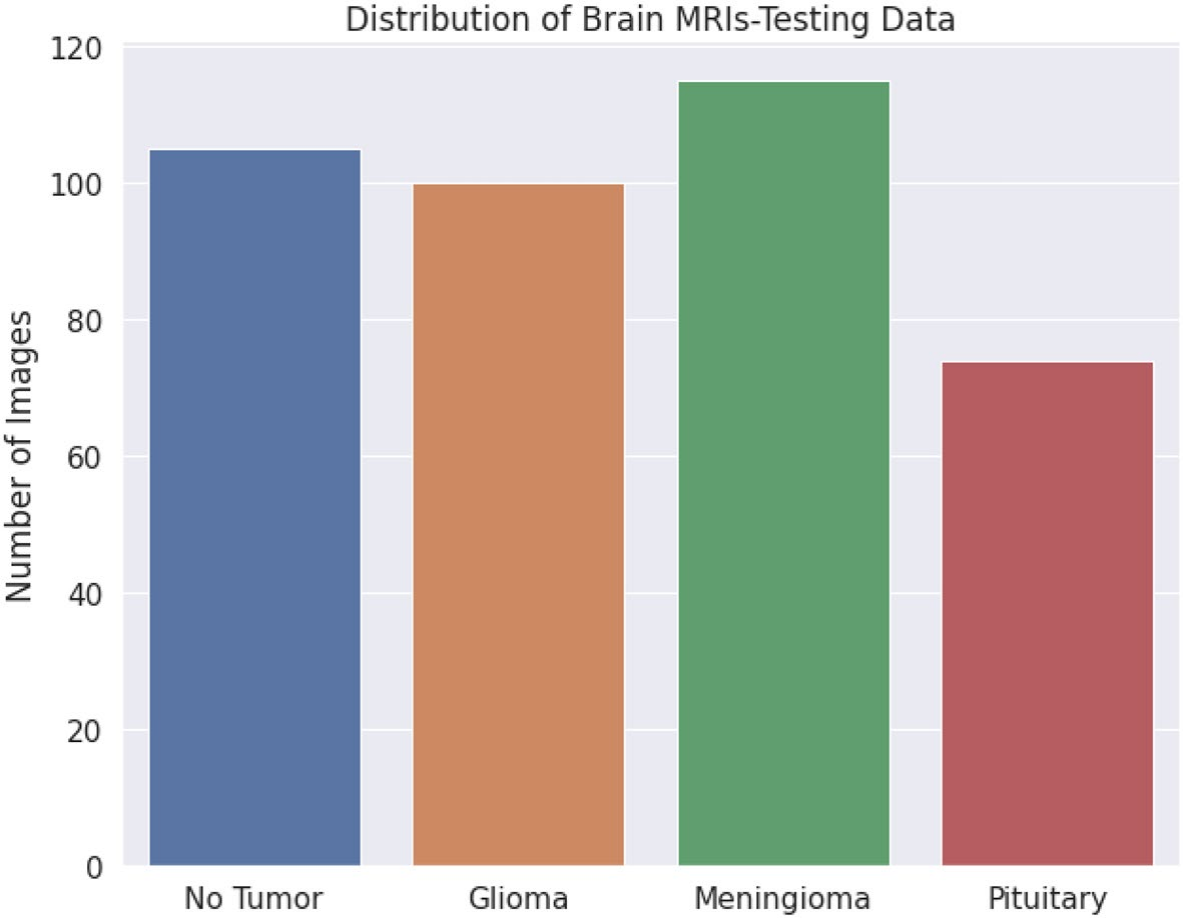
Distribution of testing data.

**Figure 12:**
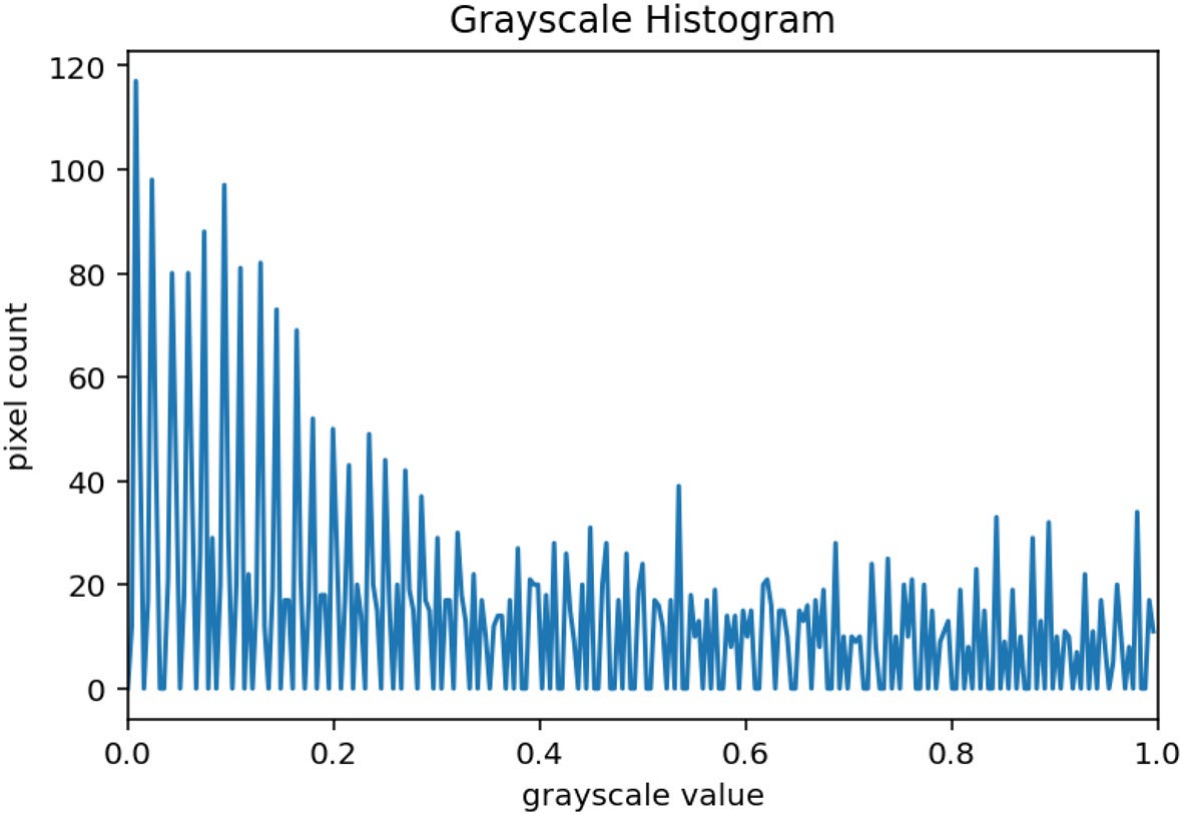
Grayscale histogram.

Upon doing the image analysis on the dataset, the following are data distributions of training and testing data as shown in Figs. 10, and 11 respectively. It was found that out of 2700 training images, 777 images belonged to Glioma tumor, 372 images belonged to no tumor category, 773 images belonged to meningioma category and 778 images belonged to pituitary tumor category. This means that the dataset is quite balanced and hence further balancing techniques like cost sensitive techniques or sampling might not be needed. The grayscale histogram distribution of an image has also been plotted below in Fig. 12.

As the MRI images are subject to noise due to Magnetic Radiation, filtering the noise is important. We can have different filters for removing the noise like band pass or Chebyshev filter but the one that we will be using is Gaussian filter that can remove the noise with a good SNR (Signal to noise Ratio)^40^ The Gaussian (or normal) distribution in univariate form has the following equation:

1 $$ G(x) = \frac{1}{{\sqrt {2\pi \sigma } }}e^{{ - \frac{{x^{2} }}{{\sigma^{2} }}}} $$

However, while working with images, we need to apply two dimensional Gaussian Filter, the equation is as follows:

2 $$ G(x,y) = \frac{1}{{\sqrt {2\Pi \sigma^{2} } }}e^{{ - \frac{{x^{2} + y^{2} }}{{2\sigma^{2} }}}} $$

The Gaussian filter was applied to the images for removing noise and smoothening the images. The Gaussian filter is a type of low-pass filter that is non-uniform. Its kernel coefficients decrease in magnitude as the distance from the center of the kernel increases. The pixels in the center of the kernel have a higher weight compared to those at the edges. Increasing the value of σ results in a wider peak and more blurring. To maintain the Gaussian nature of the filter, the kernel size must increase as σ increases. The coefficients of the Gaussian kernel depend on the value of σ, and at the edge of the kernel, the coefficients must approach zero.

**Figure 13:**
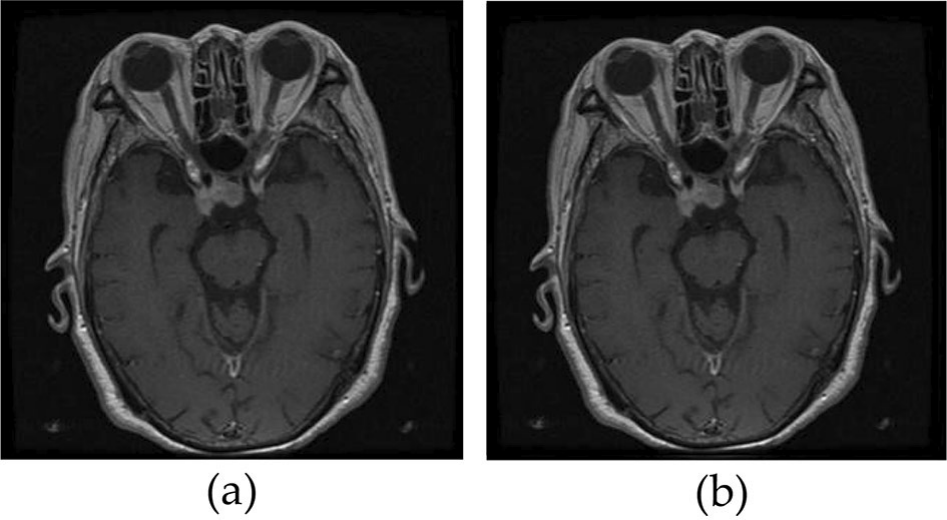
(**a**) Before applying Gaussian filter (**b**) After applying Gaussian filter.

The Gaussian filter kernel is symmetric in all directions and does not have a preference for any particular direction. It can be separated into two 1D kernels which enables faster computation. However, the use of Gaussian filters may result in a loss of image brightness. The following Fig. 13, shows the images before and after applying Gaussian Filter.

To better train the Convolutional Neural Network, we use data augmentation using Image Data Generator. The Image Data Generator object does the following: rescaling the images, zooming in for the tumor location, flipping horizontally with filling mode set to “nearest” for generating new images. Followed by data augmentation, there is also a need for transforming the training and testing images using standard transforms which do the following: horizontal Flip, Random vertical flip, Adjusting the sharpness and normalizing the image. The number of images after augmentation is 5108. After normalizing the images, it is important to convert the 3 channeled images to a single channel Gray scale images for creating a single dimension Adjacency matrix for the images. The images are converted into Grayscale values which are normalized again so that the pixel values lie in the range of (0, 1) only.

**Figure 14:**
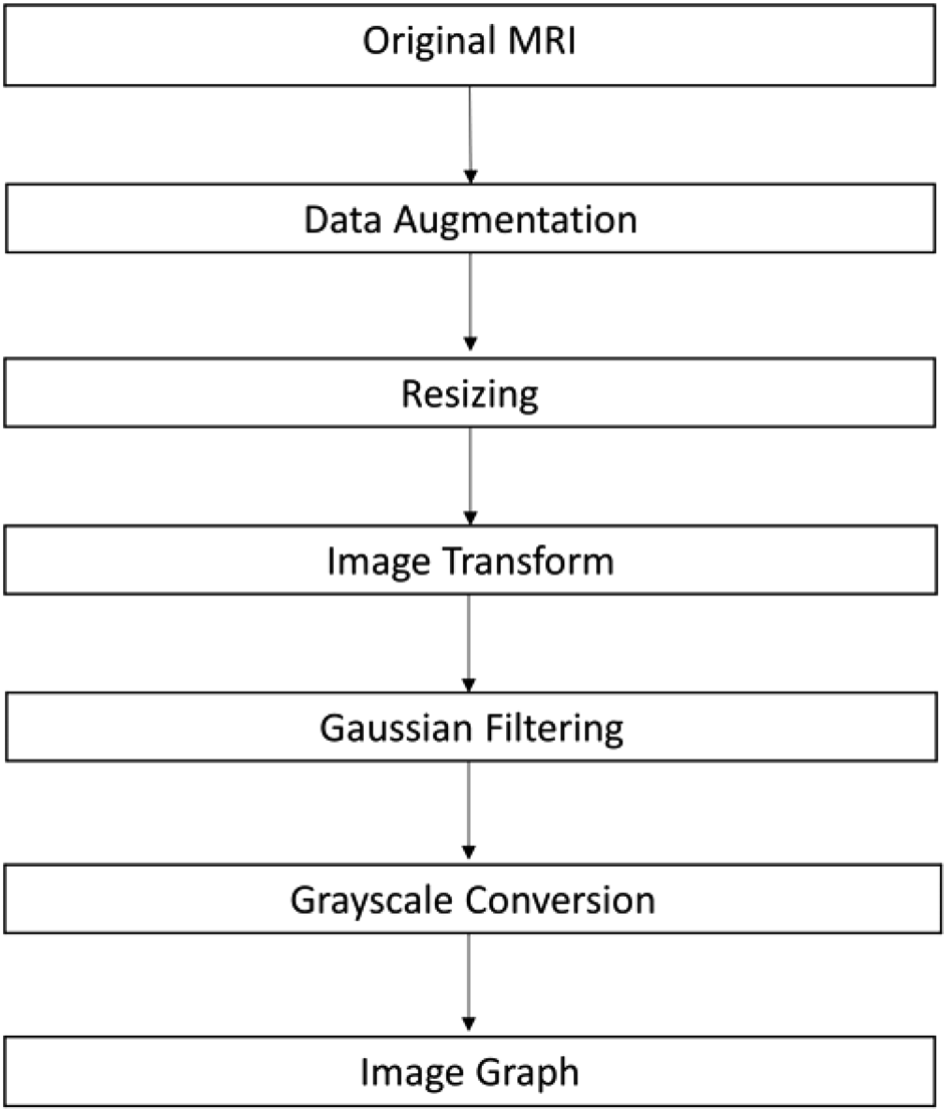
The pre-processing pipeline.

After this, to keep the model under computational constraints, we resize the images to an Image size of 30X30. The entire image pre-processing process has been shown in the Fig. 14.

After pre-processing the data, this data can be transformed using kernel superposition. These needs creating a pre-computed adjacency matrix kernel whose dimensions are compatible with our image matrix.

#### Computing a standard adjacency matrix

An adjacency matrix, denoted by A, is a way to represent a graph with all its edge-based connection. A graph G can be represented fully with a matrix-based representation where the values are either 0 or 1 depending upon whether a connection exists between two nodes or not^35^. As our model considers images as graphs, there comes a need to simplify the graph traversal order and devise a way to represent such dense connections.

**Figure 15:**
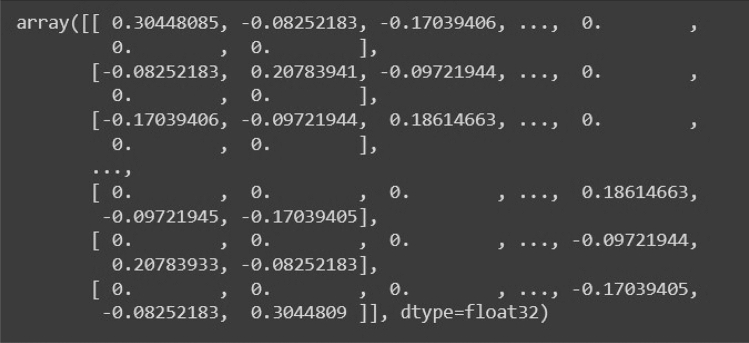
Standard weighted adjacency matrix for Net-2.

**Figure 16:**
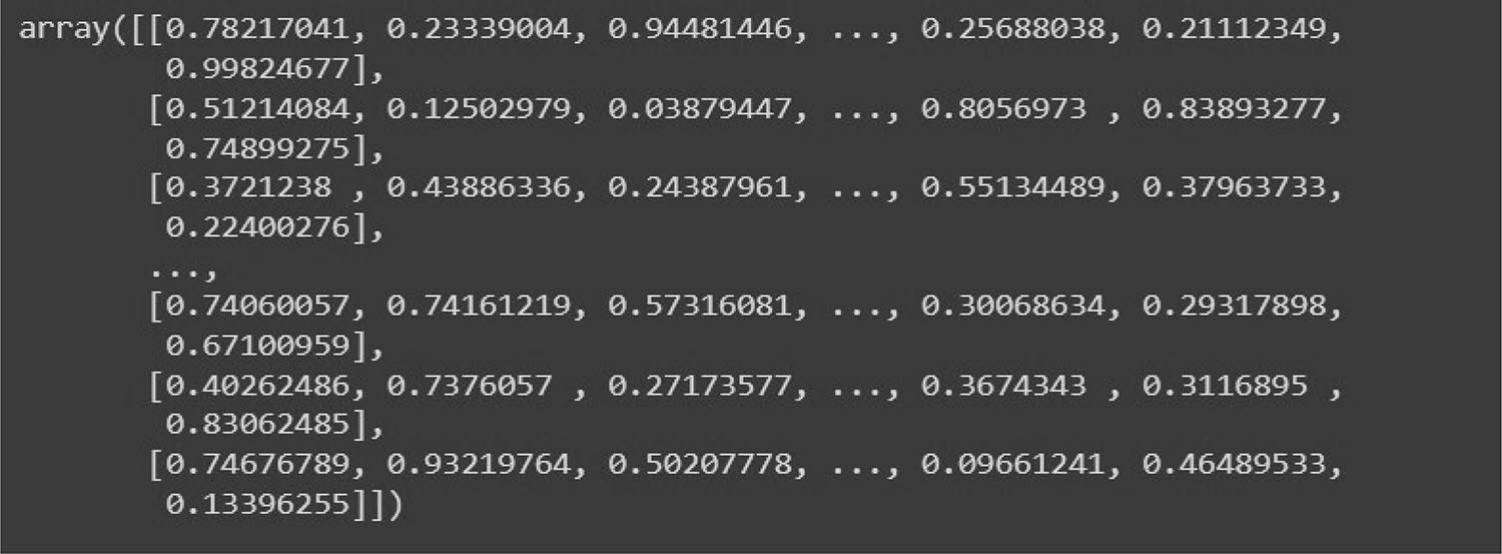
Standard uniform adjacency matrix for Net-3.

**Figure 17:**
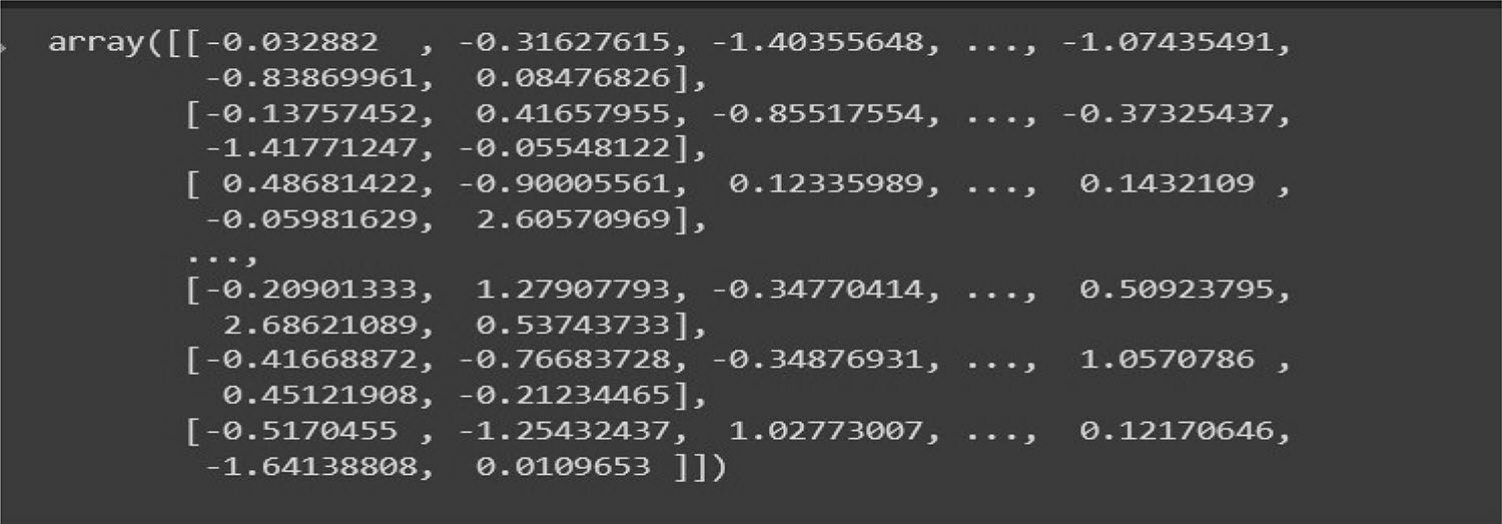
Standard log-normal adjacency matrix for Net-4.

To solve this problem, we propose using a pre-computed adjacency matrix kernel which will be superimposed on the input image matrices to generate a modified graph-based representation. This step converts an image to a weighted graph as we multiply the image vectors with this standard weighted adjacency matrix. This Adjacency matrix is calculated by projecting standard Gaussian distribution onto an n x n matrix where n represents the size of the image. Equation (1) represents Gaussian distribution for calculating the Adjacency Matrix for Net-2. After using Gaussian distribution on this matrix, we get a standard weighted Adjacency matrix that we can multiply with the Image data matrix, shown in Figs. 15, 16 and 17 respectively.

For Net-3 and Net-4, we use Uniform and Log—normal based Adjacency matrices.

#### Graph convolution

**Figure 18:**
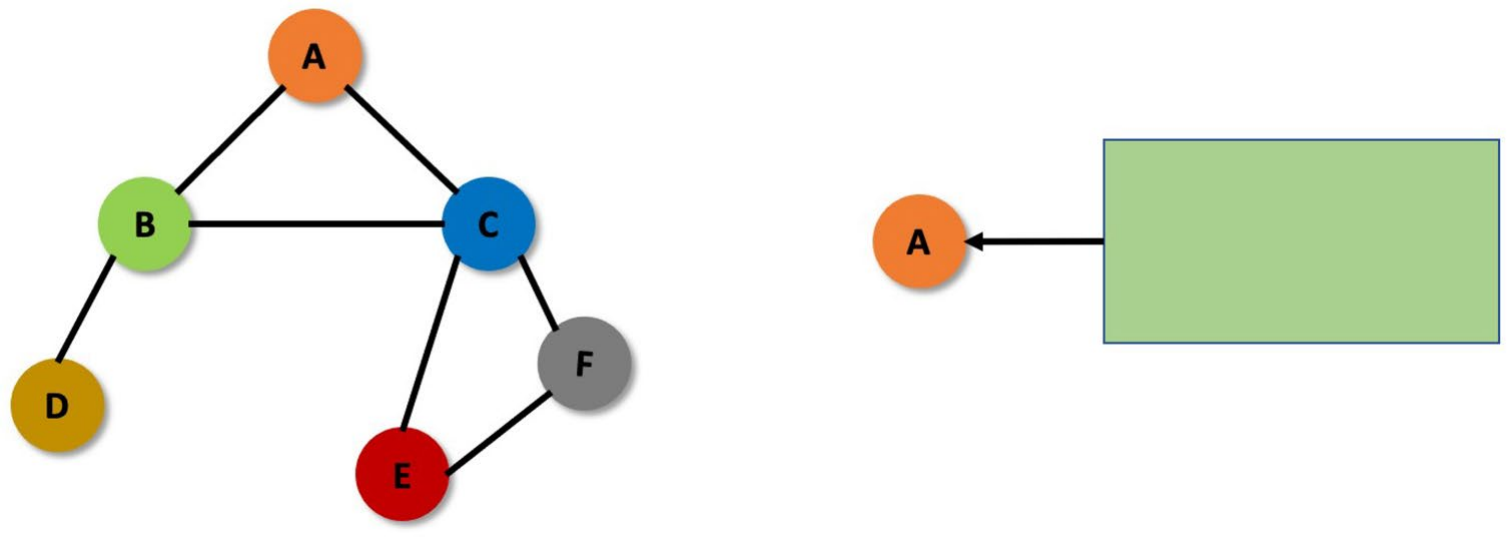
Input graph.

After multiplying the Adjacency matrix with the input images, we need to further instil the relational awareness in the present graph. To do so we introduce the concept of averaging of neighbors that is every node is updated as the average of its neighboring node, consider the following graph (Fig. 18).

Node A is the target node for which the embeddings will be created.

Here, the nodes which are neighbors to node A are B and D.

Node A will receive information from nodes B and D and then update its value. Generically stating,

3 $$ {\text{Hu }} = {\text{ UPDATE}}^{{({\text{k}})}} ({\text{Hu}}^{{({\text{k}})}} ,{\text{ AGGREGATE}}^{{({\text{k}})}} \left( {\{ {\text{hv}}^{{({\text{k}})}} ,{\text{ for all v belonging to N}}\left( {\text{u}} \right)\} } \right) $$

where, H = node features/embeddings, k = number of hops or the node number.

#### Convolutional neural network

**Figure 19:**
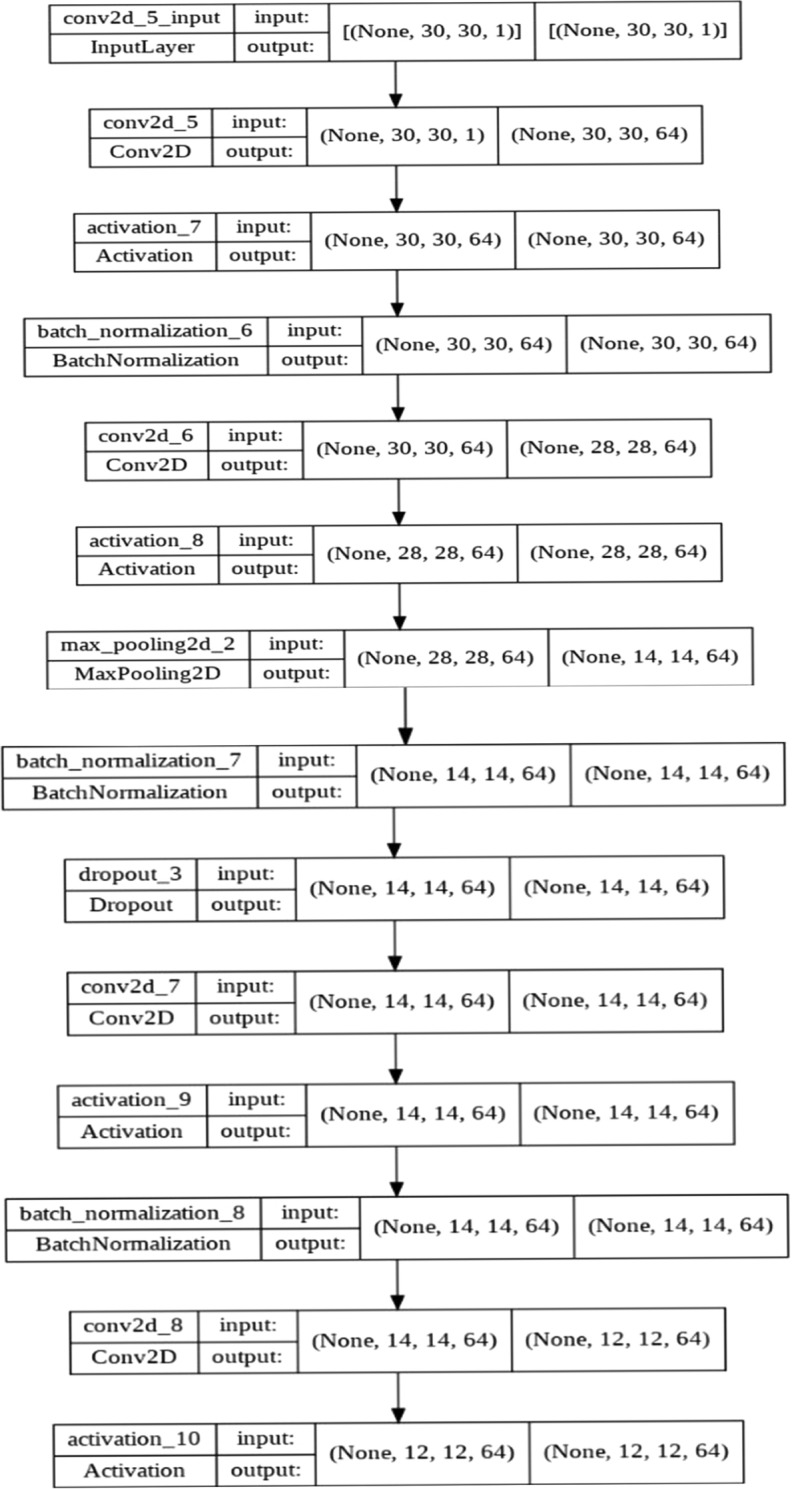
26-layered CNN architecture.

The previous step gives us a weighted graph where each node is an average of its nearest pixel neighbors. This graph is now relationally aware and can be now passed through a vanilla Convolutional Neural Network. We have taken a 26 layered Convolutional Neural Network with the following layer structure shown in Fig. 19, The convolutional neural network has alternative layers of convolution layer, with max pooling and batch normalization layers. Besides these layers, there are 2 dropout layers to enhance the performance. The detailed advantages of these layers are mentioned below:

1. *Effect of Graph Data Structure* Converting the image vector to a graph data structure helps incorporating the relational awareness in the model which yields better results in terms of both training and testing accuracy as well as a very good precision, recall, specificity and sensitivity score as the model is well adjusted on the data and is well aware of all the relational intricacies.
2. *Effect of Convolutional Neural Network with Graph Convolution* Using conventional Convolution on Graph data structure is not possible as there comes a problem of traversal order. Therefore, there comes a need for an alternative to the normal convolution operator such that this alternative is independent of the order. We perform averaging operator over the neighbors which can be placed as Graph Convolution and then it can feed into as Convolutional Neural Network normally.

## Result and discussion

In this section, we will present and analyze the experimental results obtained from the model and the five networks we have developed. The networks were trained successfully, and their performance was evaluated using different metrics, including Confusion Matrix, Accuracy, Precision, and Recall. In addition, other performance scores, such as accuracy and precision, were evaluated for informational purposes.

### Confusion matrix

An effective technique for assessing a classification algorithm's effectiveness is a confusion matrix. It consists of four sections, where the top left corner represents the true positives, which are the instances where the algorithm correctly predicted a positive output.

**Figure 20:**
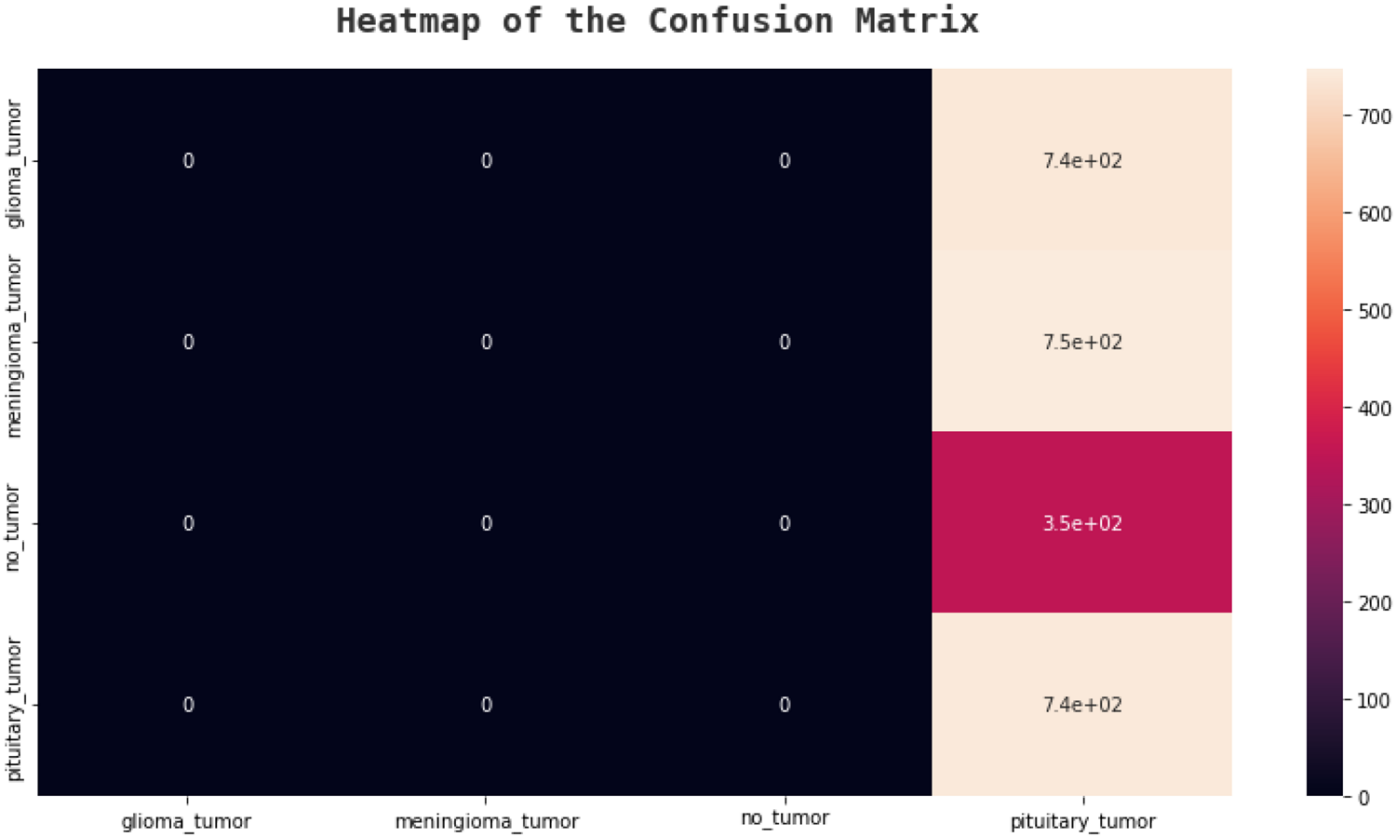
Net-1 confusion matrix.

**Figure 21:**
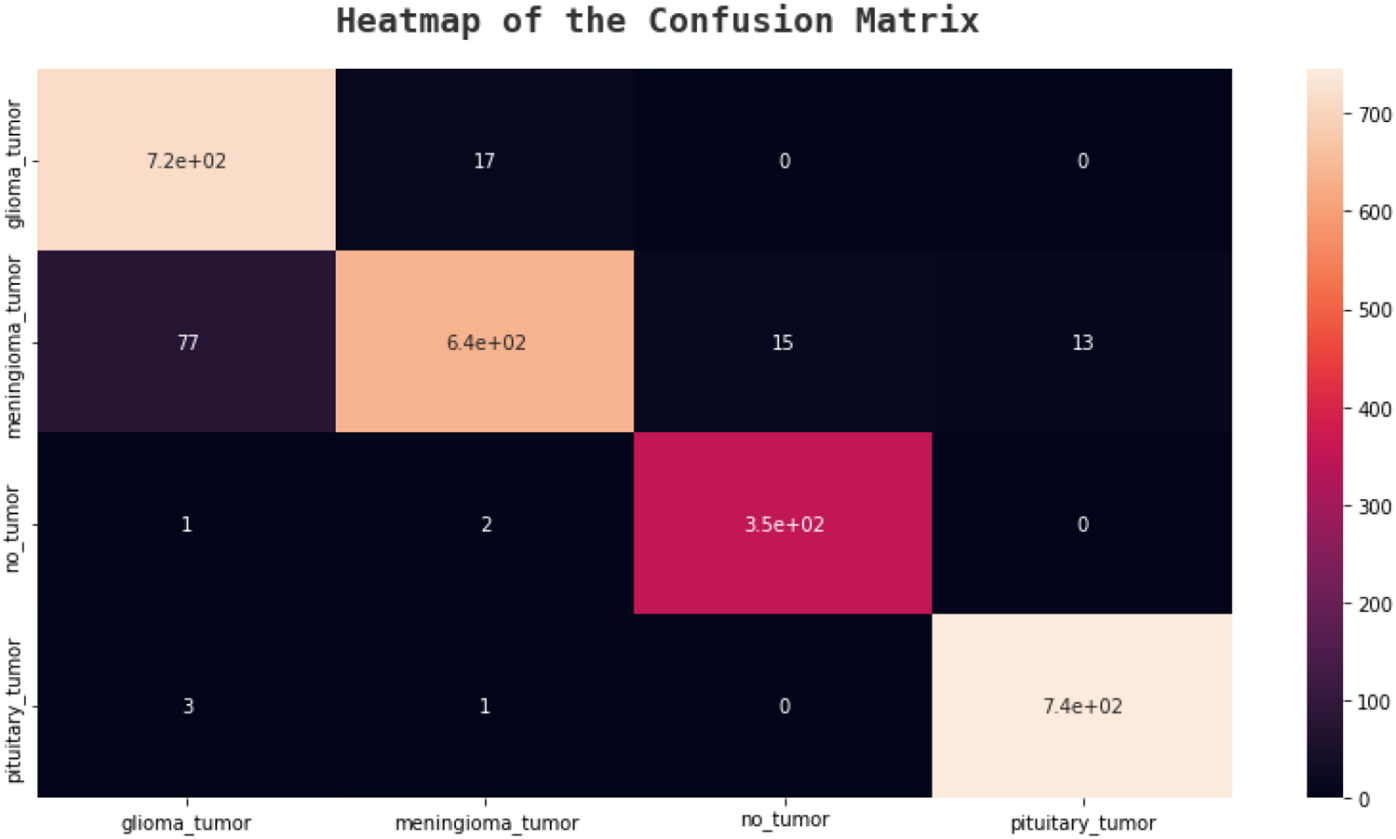
Net-2 confusion matrix.

**Figure 22:**
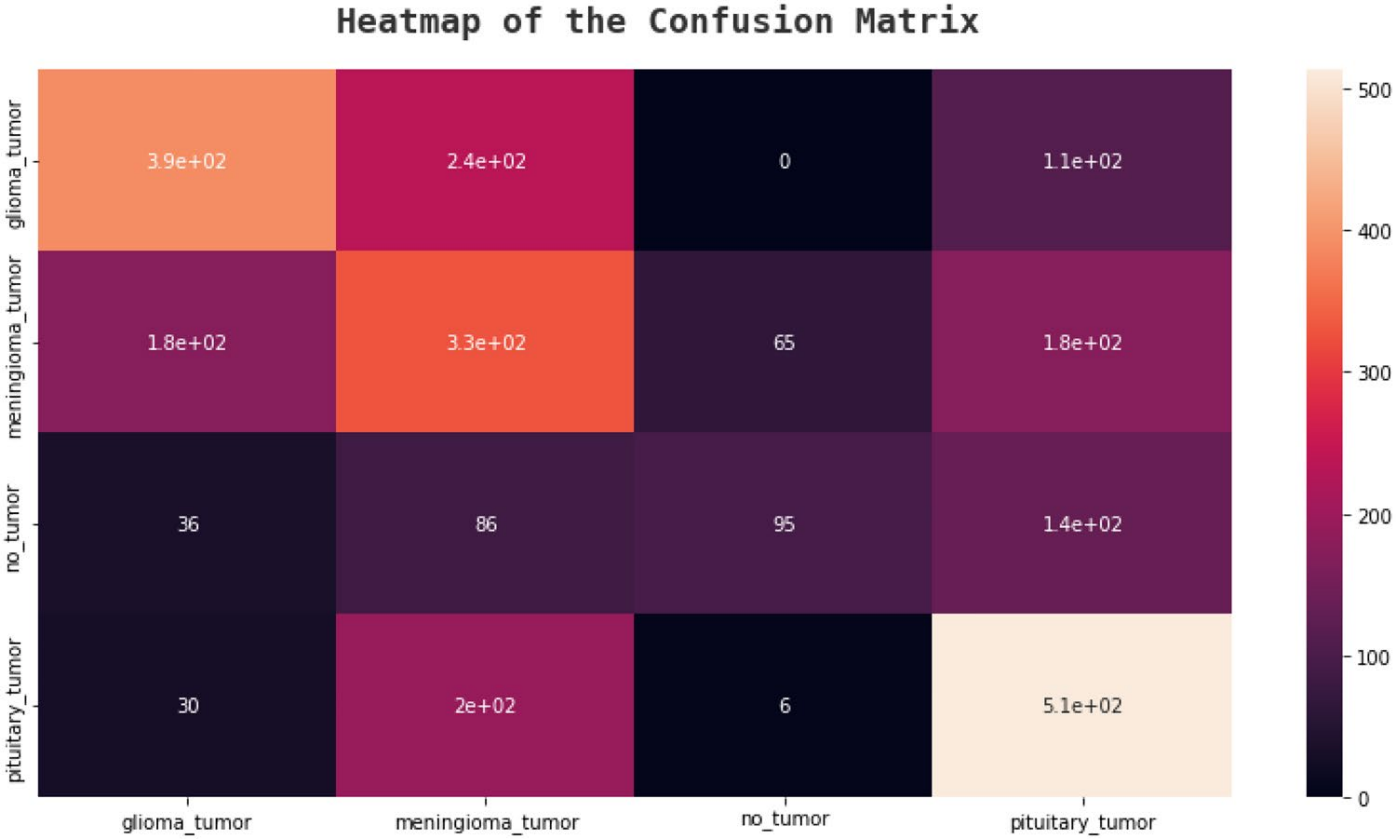
Net-3 confusion matrix.

**Figure 23:**
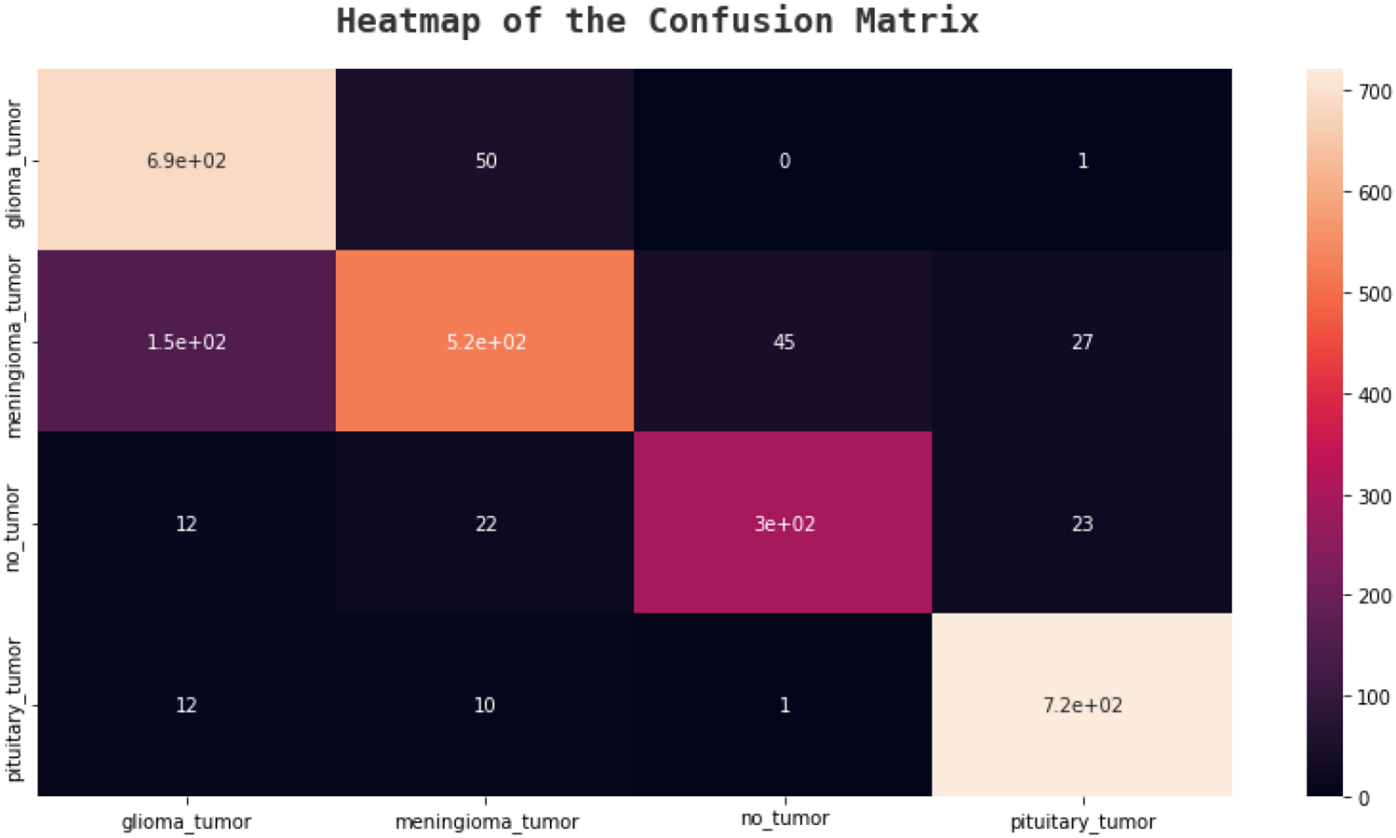
Net-4 confusion matrix.

The top right corner represents the false positives, which are the instances where the algorithm predicted a positive output, but the actual output was negative. The bottom left corner represents the false negatives, which are the instances where the algorithm predicted a negative output, but the actual output was positive. The bottom right corner represents the true negatives, which are the instances where the algorithm correctly predicted a negative output^41^. The following figures, Figs. 20, 21, 22, and 23 show the confusion matrices corresponding to various networks Net 1, Net 2, Net 3, and Net 4 Respectively.

### Accuracy

Accuracy is one of the most popular measures for evaluating classification methods. and it measures how often the model correctly predicts the class of an observation out of all the observations as shown in Eq. (4). The accuracy score along with the other metrics are mentioned in Table 1.

4 $$ \frac{True\;Positive + True\;Negative}{{True\;Positive + True\;Negative + False\;Positive + False\;Negative}} $$

**Table 1:** Notation and their corresponding metrics.

Notation	Metrics
a1.1	Precision for Class 0
a1.2	Precision for Class 1
a1.3	Precision for Class 2
a1.4	Precision for Class 3
a2.1	Recall for Class 0
a2.2	Recall for Class 1
a2.3	Recall for Class 2
a2.4	Recall for Class 3
a3	Testing accuracy
a4	Average testing accuracy

### Precision

Actually, The ratio of true positives to the total number of positive predictions is the precision value for a classification algorithm. as shown in Eq. (5).

5 $$ \frac{True\;Positive}{{True\;Positive + False\;Positive}} $$

### Recall

A classification algorithm's recall value is determined by the proportion of true positives to all positives, as shown in Eq. (6).

6 $$ \frac{True\;Positive}{{True\;Positive + False\;Negative}} $$

### Loss curve

**Figure 24:**
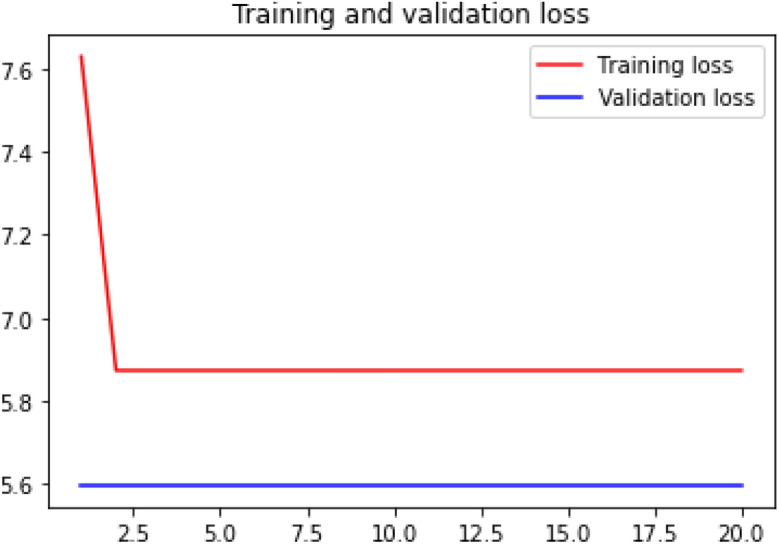
Net-1 loss curve.

**Figure 25:**
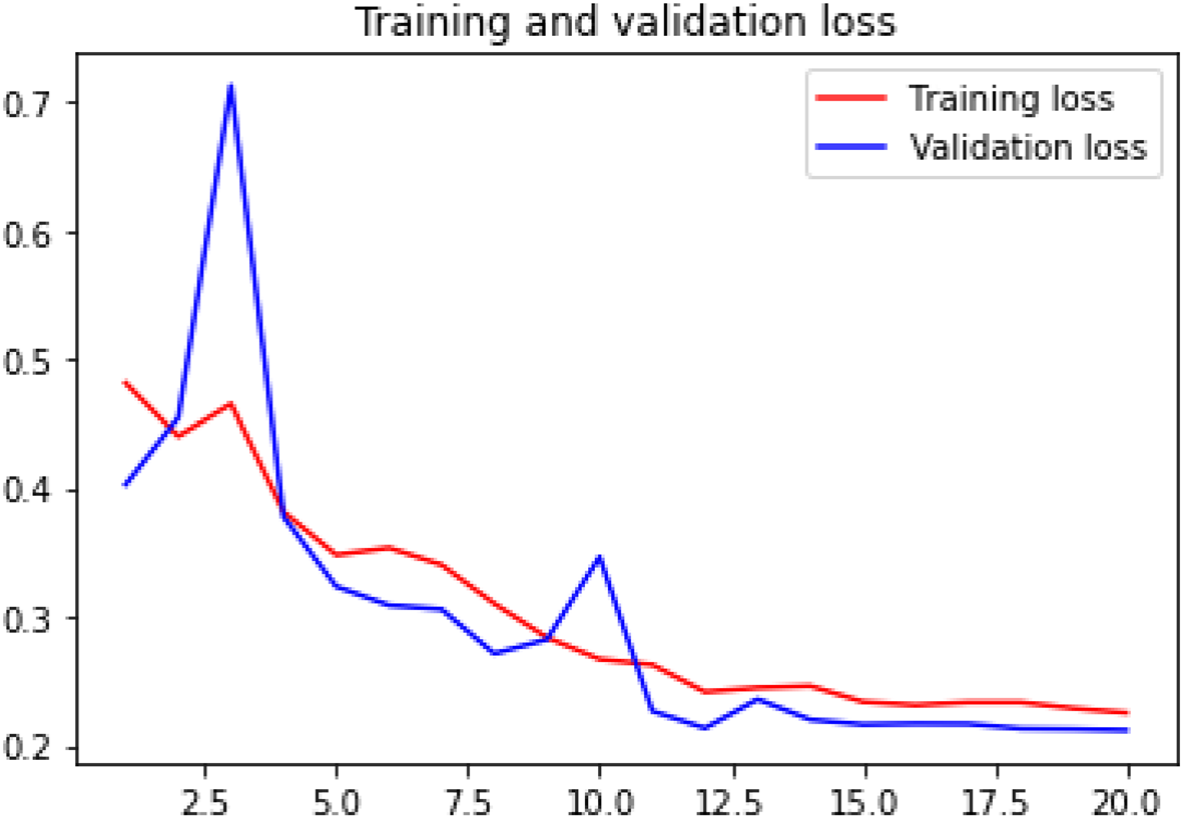
Net-2 loss curve.

**Figure 26:**
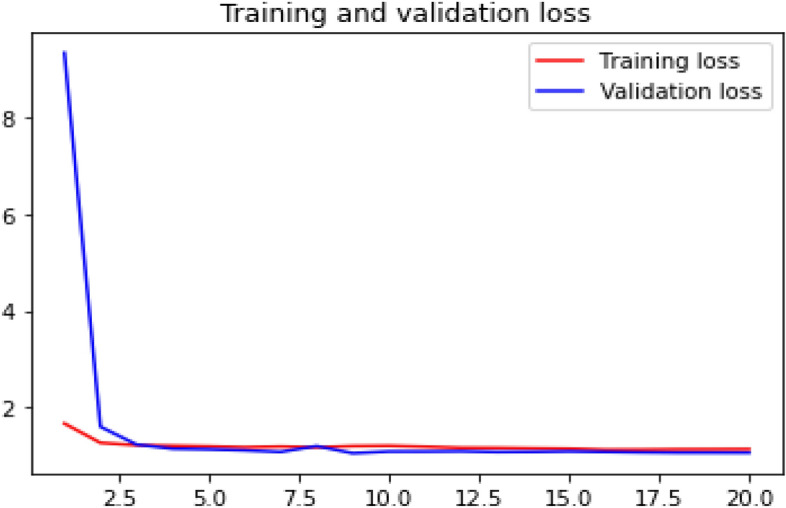
Net-3 loss curve.

**Figure 27:**
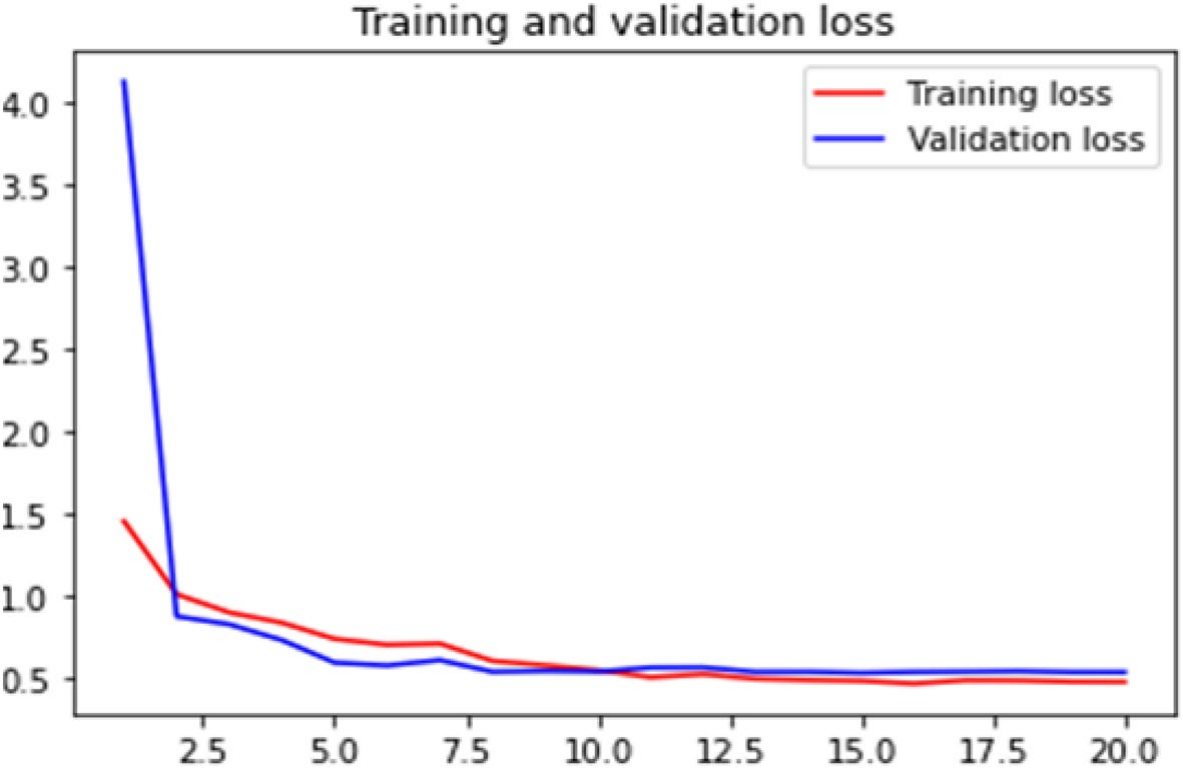
Net-4 loss curve.

Loss curve of a training process shows the change in the value of loss function over the epochs. The loss curve for our model training is presented in Figs. 24, 25, 26 and 27.

### Accuracy curve

**Figure 28:**
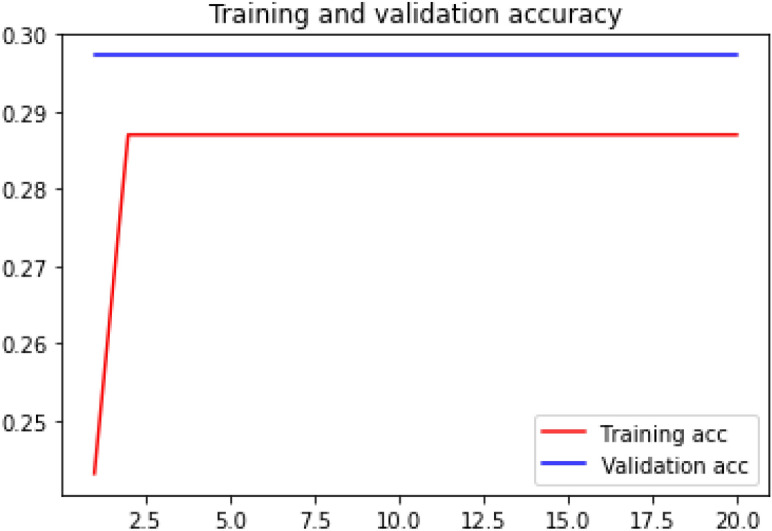
Net-1 accuracy curve.

**Figure 29:**
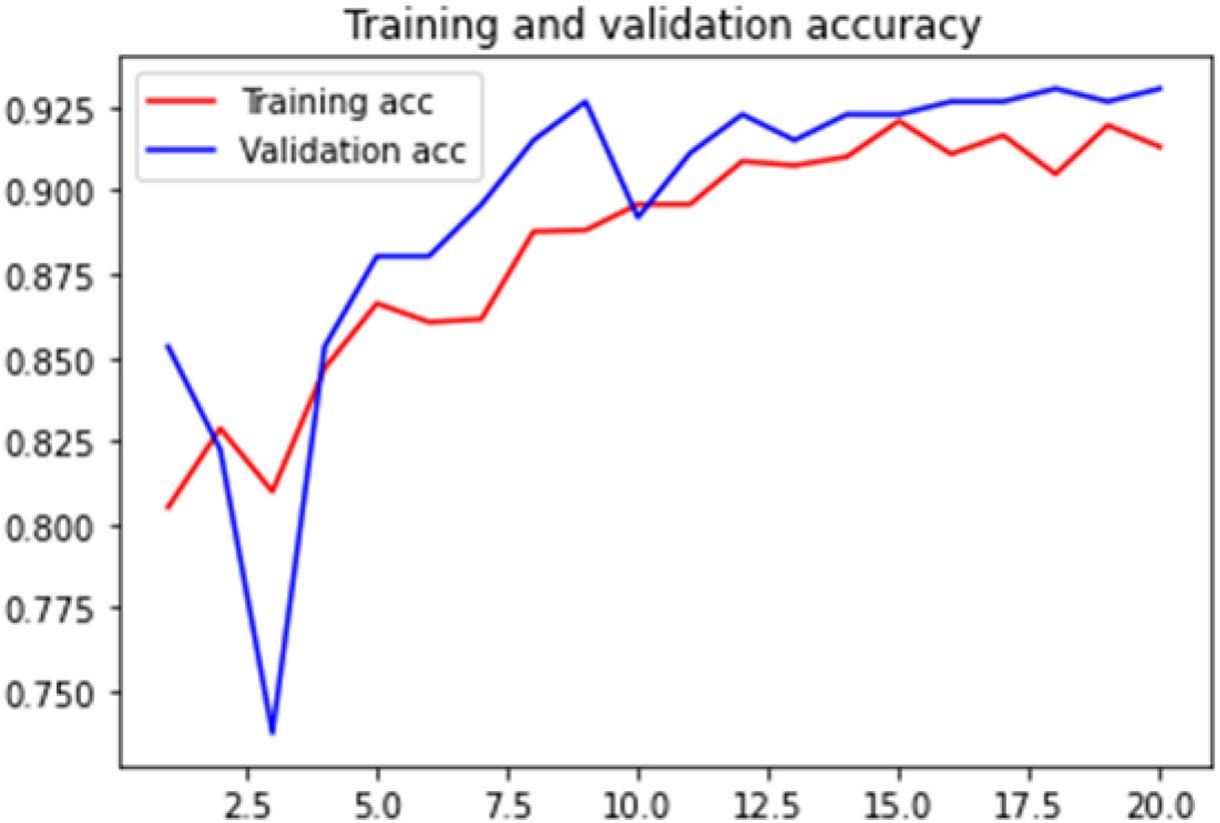
Net-2 accuracy curve.

**Figure 30:**
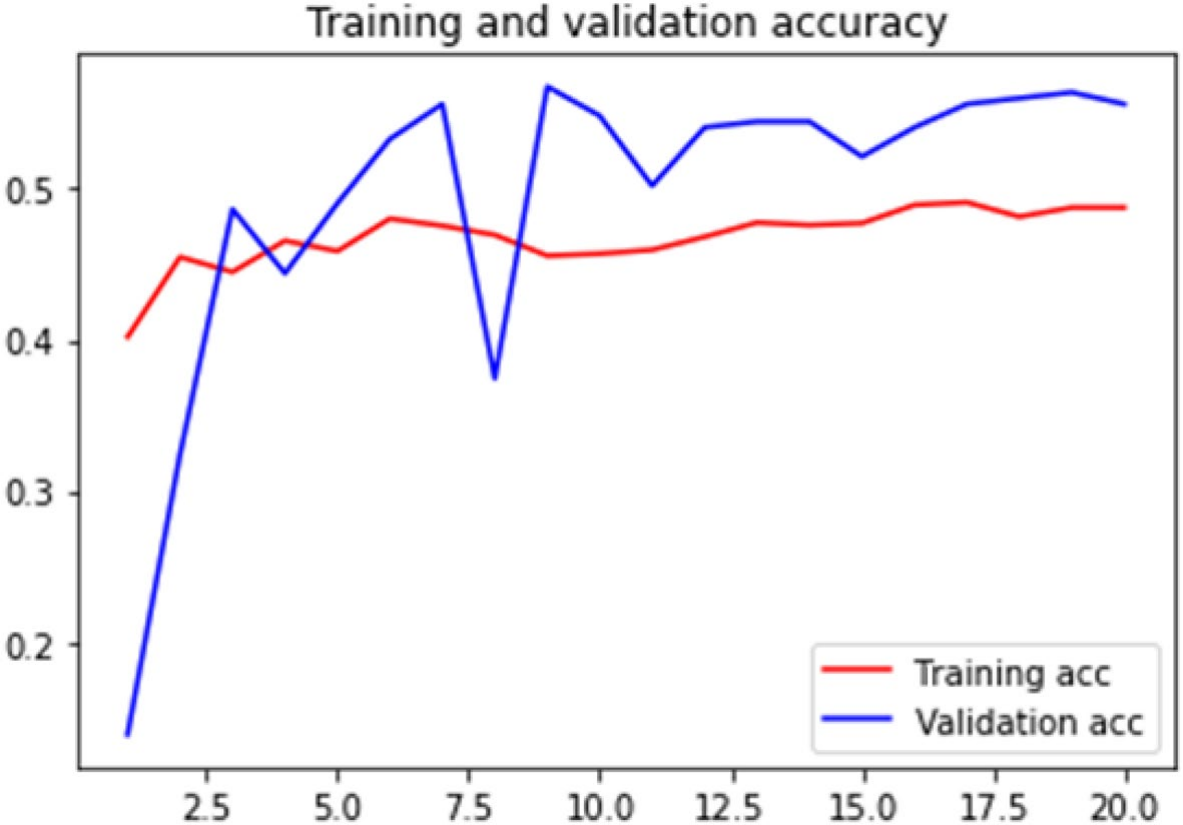
Net-3 accuracy curve.

**Figure 31:**
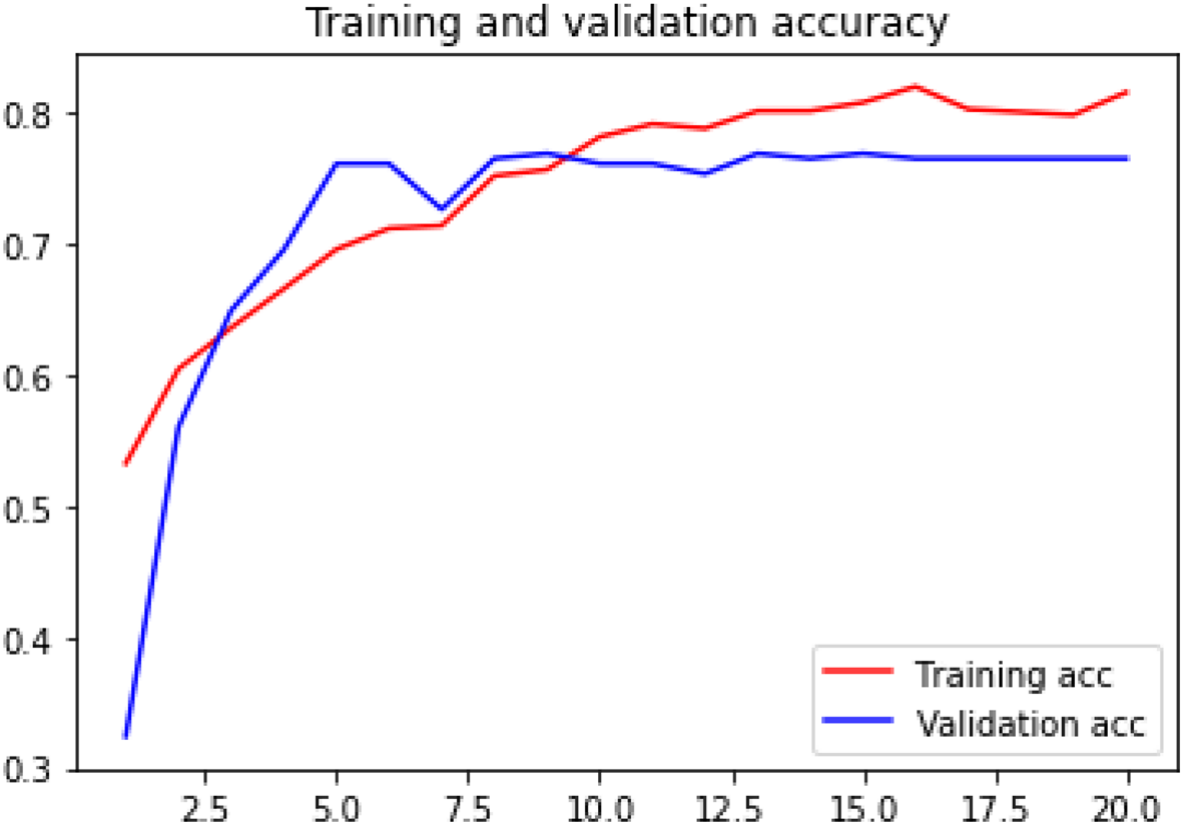
Net-4 accuracy curve.

Loss curve of a training process shows the change in the value of loss function over the epochs. The loss curve for our model training is presented in Figs. 28, 29, 30 and 31.

Accuracy curve shows the change in value of the accuracy with epochs. A detailed performance report is shown in Table 1 for all the networks.

By looking at comparison between all the proposed Networks (Net-0, Net-1, Net-2, and Net-3), we can conclude that the performance of Net-2 is good compared to remaining Networks, shown in Table 2.

**Table 2:** Comparison between the networks.

Metric	Different networks				
	Net-0	Net-1	Net-2	Net-3	Net-4
a1.1	0.9	0	0.9	0.62	0.8
a1.2	0.89	0	0.97	0.39	0.86
a1.3	0.9	0	0.96	0.57	0.87
a1.4	0.87	0.29	0.98	0.55	0.93
a2.1	0.91	0	0.98	0.53	0.93
a2.2	0.88	0	0.91	0.44	0.7
a2.3	0.85	0	0.98	0.27	0.84
a2.4	0.94	1	0.99	0.69	0.97
a3	0.92	0.25	0.95	0.48	0.87
a4	0.92	0.29	0.95	0.51	0.87

In Table 3, we have compared the accuracy of our proposed network (Net-2) with the state-of-the-art methods.

**Table 3:** Comparison with state-of-the-art techniques.

Existing methods	Average testing accuracy (%)
Sudharani and Sarma^18^	89.20
Nabizadeh et al.^19^	91.50
Subashini et al.^20^	91.00
Nabizadeh and Kubat^22^	79.3 ± 0.3
Proposed method (Net-2)	95.01

## Conclusions

In this paper, a novel CNN based GNN model is proposed to predict whether a person has brain tumor or not and if yes then which type (Meningioma, Pituitary or Glioma). The proposed Graph Convolutional Neural Network model considers non-Euclidean distances in image data and achieved an accuracy of 95.01%. Various networks (Net-0, Net-1, Net-2, Net-3 and Net-4) were trained, and it was found that Net-2 with Graph input-based CNN having DO and BN with Gaussian Adjacency matrix achieves the highest accuracy of 95.01%. With the current performance our proposed model stands as a vital alternative for the Statistical Detection of Brain Tumor in suspected patients.

## Data Availability

The dataset we used is a Public Dataset. The datasets generated and/or analyzed during the current study are available in the Brain Tumor Classification Dataset repository, Kaggle https://doi.org/10.34740/KAGGLE/DSV/1183165.
